# Biomimetic Multiscale‐Structured Biomass Graphene/Polyurethane Sponge Composite for Flexible Pressure Sensors and Intelligent Cushioning Materials

**DOI:** 10.1002/advs.202517511

**Published:** 2025-11-25

**Authors:** Runmin Xu, Pusen Cao, Tingting Zhang, Jie Wei, Yue Wang, Zhi Huang, Zhenghang Chen, Jianfeng Xi, Yong Guo, Yuxia Chen

**Affiliations:** ^1^ College of Materials and Chemistry Anhui Agricultural University Hefei 230036 China; ^2^ Anhui Healthy Sleep Home Furnishings Engineering Research Center Hefei 230036 China; ^3^ Anhui Le Fu Er Home Technology Co. Ltd Hefei 230036 China; ^4^ Key Laboratory of Wood and Bamboo Furniture National Forestry and Grassland Administration Changsha Hunan 410000 China

**Keywords:** biomass graphene, conductive sponge, flexible sensor, multiscale structure, sleep

## Abstract

The development of furniture cushioning materials that combine excellent mechanical properties with sensing capabilities is essential for non‐intrusive, long‐term health monitoring. This study presents a multifunctional conductive sponge (MAPU) that synergistically integrates the macroscale mechanical support of a polyurethane (PU) sponge with the microscale sensing characteristics of aerogels through bionic multiscale structural design. Biomass‐derived graphite nanoflakes serve as the conductive units to in situ construct a 3D interpenetrating aerogel network on the PU sponge skeleton. This unique heterogeneous structure retains the flexibility and elasticity of PU sponge while providing exceptional piezoresistive sensing performance, including high sensitivity (0.821 kPa^−1^ in the 0–53 kPa range), a wide response range (up to 242 kPa), fast response time (≤ 50 ms), and outstanding cycling stability (> 30 000 cycles). Equally important, MAPU also demonstrates washability, flame retardancy, breathability, and sound absorption, making it practical for household applications. An intelligent mattress composed of a MAPU sensor array enables real‐time monitoring and precise recognition of sleep postures, along with bedsore risk alerts. This work offers a high‐performance, multifunctional, and high‐safety core material solution for advanced smart home technologies and continuous health monitoring systems.

## Graphical abstract

In this study, a multifunctional conductive sponge composite (MAPU) is developed using a biomimetic, hierarchical construction strategy emulating the growth process of natural wood. This approach involved integrating biomass‐derived graphitic nanoflakes, synthesized from luffa vine (LVGN), into a polyurethane (PU) sponge matrix. The resulting MAPU composite is subsequently fabricated into functional sensors and successfully deployed for sleep monitoring and health‐risk alert applications.

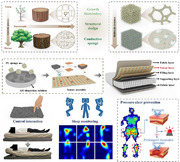

## 1 Introduction

Sleep, a fundamental physiological rhythm essential for sustaining human life and health, is inextricably linked to physical and mental well‐being.^[^ ^1^ ^]^ With the growing integration of smart home technologies and holistic health concepts, seamlessly integrating advanced sensing technologies into sleep products to develop intelligent bedding that combines high comfort with sensing capabilities has become a pivotal research direction.^[^ ^2^ ^]^ Flexible pressure‐sensing technology, capable of non‐intrusively capturing real‐time dynamic changes in human body pressure distribution, has garnered significant attention. It provides the core data foundation for assessing sleep quality, issuing early warnings of potential health risks, and enabling personalized sleep intervention strategies.^[^ ^3^ ^]^

Among various flexible sensing technologies, piezoresistive sensors stand out for their simple structure, straightforward response mechanism, and excellent mechanical flexibility, demonstrating substantial application potential in electronic skin, wearable medical devices, and human–machine interfaces.^[^ ^4^, ^5^ ^]^ Although significant progress has been made in enhancing sensitivity and expanding linear response ranges, long‐term practical application in sleep products remains challenging.^[^ ^6^, ^7^ ^]^ Sensing materials generally struggle to simultaneously achieve high sensing performance and sufficient mechanical strength, compromising stability under complex, prolonged mechanical stresses exerted by the human body. Intricate fabrication processes and high raw material costs also hinder their widespread adoption.^[^ ^8^ ^]^ Additionally, most sensors lack essential multifunctional properties suitable for home products, such as washability, breathability, flame retardancy, and skin‐friendly comfort, severely constraining their practical application potential.^[^ ^9^ ^]^

Sponge, an indispensable cushioning material in soft furnishings, is an ideal substrate for constructing intelligent sensing bedding because of its superior elasticity, excellent biocompatibility, and outstanding stress‐dispersing capability.^[^ ^10^, ^11^ ^]^ Developing high‐performance conductive networks on commercial sponge substrates enables combining comfort with sensing functionality. The mainstream approach primarily employs dip‐coating highly conductive materials, such as carbon‐based materials,^[^ ^12^, ^13^ ^]^ conductive polymers,^[^ ^14^ ^]^ Mxene,^[^ ^15^ ^]^ or perovskite‐derived oxides,^[^ ^16^ ^]^ onto polyurethane (PU) sponge skeletons to form conductive networks. However, this strategy typically encounters two major challenges. First, the fragile interfacial bonding between conductive fillers and the sponge matrix leads to poor structural stability under complex stress–strain conditions, causing signal attenuation or failure.^[^ ^17^ ^]^ Second, the inherent stress plateau effect of sponges causes stresses to remain nearly constant over a specific compressive strain range, creating detection dead zones that severely limit response range and sensitivity.^[^ ^18^, ^19^ ^]^

Overcoming these limitations requires synergistic optimization of both mechanical and sensing properties. Multiscale structural design presents a promising solution. Inspired by the hierarchical structure of natural wood, this study constructs an in situ formed, inherently rigid conductive aerogel network within the macroporous structure of the sponge. The microscale network serves a dual function: first, it acts as a secondary support structure, effectively suppressing the stress plateau effect of the sponge and broadening the stress response range of the sensor. Second, its intricate 3D conductive pathways significantly enrich electrical signal transmission within the sponge pores, thereby simultaneously enhancing sensitivity and stability. Within this structural framework, biomass‐derived graphitic nanoflakes serve as ideal conductive elements for this multiscale network owing to their excellent electrical conductivity, high specific surface area, and sustainable sourcing.^[^ ^20^ ^]^ Agricultural waste luffa vine (LV) represents a potential biomass resource. Its utilization can mitigate environmental pollution caused by waste disposal and reduce the reliance of functional carbon materials on non‐renewable resources, thereby enhancing the sustainability of sensor manufacturing.^[^ ^21^ ^]^

Accordingly, this study proposes a strategy of hierarchical construction and multiscale synergy. Luffa vine was converted into high‐quality biomass‐derived graphitic nanoflakes (LVGN) through catalytic graphitization followed by wet ball‐milling. Subsequently, emulating the layer‐by‐layer growth pattern observed in wood, an LVGN conductive layer was first deposited on the PU sponge skeleton via dip coating. Subsequently, an in situ LVGN aerogel network was constructed within the sponge pores through freeze‐drying, yielding a multifunctional conductive sponge composite (MAPU) with integrated sponge and aerogel phases. We systematically investigated the exceptional sensing performance of MAPU, confirming its high sensitivity (0.821 kPa^−1^ within 0–53 kPa), broad response range (0–242 kPa), and ultra‐long cyclic compression durability (>30,000 cycles). Furthermore, as a furniture cushioning material, MAPU not only provides support and pressure relief but also exhibits essential practical functionalities such as washability, breathability, flame retardancy, and sound absorption, fully satisfying the requirements for comfort and safety in sleep products. Capitalizing on these advantages, we integrated MAPU sensor arrays into a smart mattress, successfully enabling real‐time monitoring and accurate identification of body pressure distribution and sleep postures, as well as early warnings of pressure injury (decubitus ulcer) risks. This work provides a viable technical pathway for developing flexible pressure sensors that simultaneously offer superior sensing performance, exceptional comfort, and robust safety. It holds significant promise for advancing the development and application of sophisticated sensing technologies in smart home systems and health monitoring.

## 2 Results and Discussion

### 2.1 Biomimetic Design of MAPU

**Figure 1:**
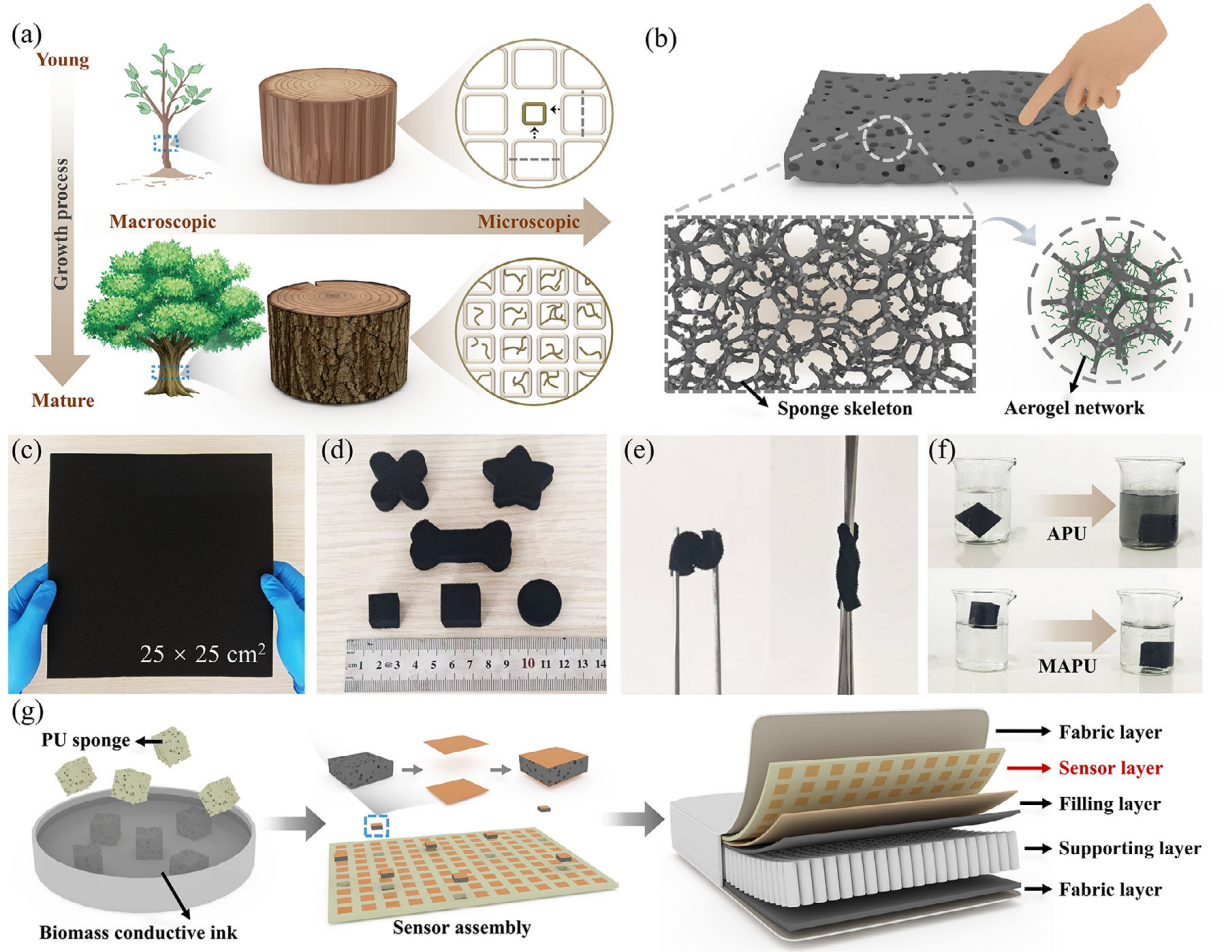
a) Schematic diagram of wood growth process; b) biomimetic design of multiscale structures; c, d) MAPU enabling customization of size and shape; e) excellent flexibility and agility of MAPU; f) comparison of soaking states of aerogel–PU (APU) and MAPU; g) process diagram for integrating MAPU sensor array into a smart mattress.

Inspired by the sophisticated hierarchical structure of naturally evolved wood, we designed a multilevel structure for the MAPU conductive sponge. Wood unifies mechanical strength and substance transport functionality through the cross‐scale synergy of its micron‐scale tracheid skeleton and nanoscale fibril network (**Figure** **1**a).^[^ ^22^ ^]^ Drawing inspiration from the microstructure of wood and its cellular growth processes, this work proposes and implements an ordered hierarchical assembly strategy.

The PU sponge skeleton serves as a micron‐scale load‐bearing framework, functionally analogous to the primary cell wall in wood, providing the composite with essential mechanical properties and a 3D morphology. Mimicking the deposition of the secondary cell wall onto the inner surface of the primary wall in wood,^[^ ^23^ ^]^ the internal 3D framework of the PU sponge was coated with a conductive layer via material infiltration and coating, establishing a primary conductive network. Subsequently, through a second infiltration step followed by freeze‐drying, a conductive aerogel network was grown in situ throughout the sponge pores onto this conductive layer. This network, reminiscent of the microtubule network within the wood cell cytoplasm, plays a dual role in structural support and transportation.^[^ ^24^ ^]^ It serves as a secondary support structure, effectively mitigating the stress plateau effect of the sponge skeleton. Simultaneously, it establishes a complex electron transport network interconnected with the primary conductive layer, forming the structural foundation for the high sensitivity of the sensor.

Finally, structural reinforcement and complete encapsulation of the entire hierarchical structure were achieved through polydimethylsiloxane (PDMS) infiltration and curing. This process is functionally similar to the lignification of the wood cell wall. It not only imparts exceptional mechanical robustness and environmental adaptability to the composite but, crucially, ensures the integrity and efficiency of the microscopic conductive networks under prolonged and complex mechanical stress. This biomimetic strategy successfully combines the elasticity and flexibility of the macroscopic sponge skeleton with the conductivity and high response sensitivity of the microscopic aerogel network, offering a novel approach for constructing flexible pressure sensors that harmonize mechanical performance with sensing capabilities (Figure 1b).

Furthermore, this biomimetic fabrication strategy exhibits high scalability and processing flexibility, enabling the batch production of conductive sponges in various sizes and arbitrary shapes (Figure 1c,d). Moreover, the MAPU composite demonstrates exceptional flexibility and environmental stability, showing no significant detachment of conductive material or structural damage even under repeated bending or prolonged water immersion (Figure 1e,f). Capitalizing on its outstanding properties, we further explored its potential for scalable application in intelligent sleep products. Specifically, MAPU was integrated into mattress structures, serving as both a flexible cushioning material and a distributed sensor network (Figure 1g). Leveraging modern mattress manufacturing processes, this integration enables smart mattresses to achieve real‐time monitoring of sleep states and deliver intelligent responses. This approach provides a pioneering material foundation and application demonstration for home‐based health monitoring.

### 2.2 Preparation and Characterization of LVGN

**Figure 2:**
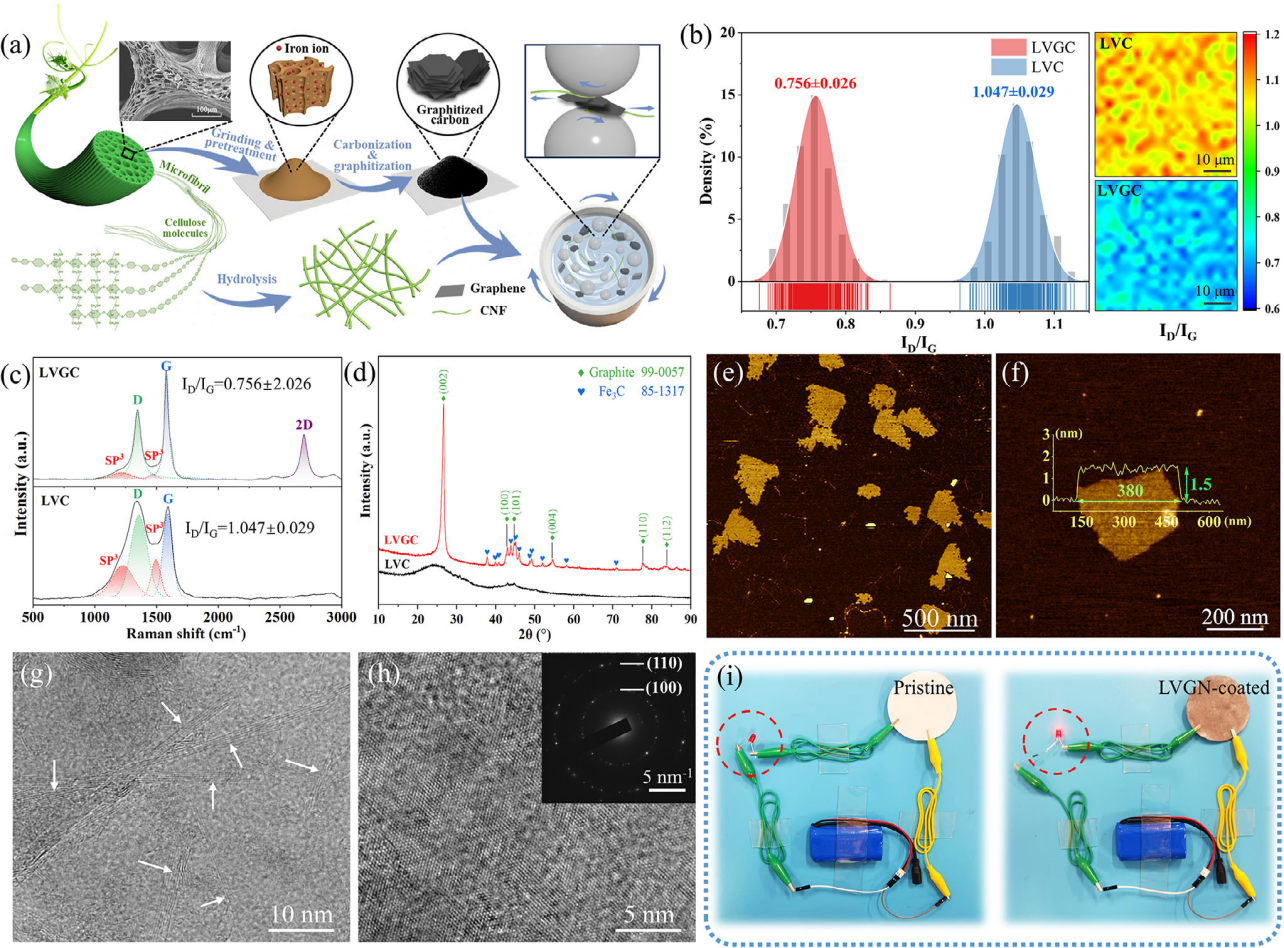
a) Preparation of biomass‐derived conductive materials; b) numerical distribution of I_D_/I_G_; c) Raman spectra and d) XRD patterns of LVC and LVGC; e, f) AFM micrographs, g) HRTEM micrographs and h) electron diffraction pattern of LVGN; i) conductivity test of LVGN/CNF coating.

The performance of flexible piezoresistive sensors critically depends on the properties of conductive materials and the architecture of conductive networks.^[^ ^25^ ^]^ To address this, we developed a strategy to produce high‐quality graphitic nanoflakes from renewable LV (**Figure** **2**a; Figure S1, Supporting Information). Instead of relying on traditional, energy‐intensive, ultra‐high‐temperature pyrolysis, highly graphitized biochar (LV‐derived graphitic carbon; LVGC) was obtained by efficiently catalyzing biomass graphitization at 1000 °C through an iron‐catalyzed dissolution–precipitation mechanism. The catalytic mechanism (Figure S2, Supporting Information) involves continuous dissolution of amorphous carbon, formed during biomass pyrolysis, into Fe, followed by precipitation into highly ordered graphite structures.^[^ ^26^ ^]^

Scanning electron microscopy (SEM) and transmission electron microscopy (TEM) graphs (Figure S3, Supporting Information) clearly revealed morphological changes in LVGC before and after acid washing. The dense, onion‐ or worm‐like graphite shell surrounding the Fe/C core transformed into a relatively loose hollow shell structure after core removal, providing an ideal precursor for subsequent mechanical exfoliation.^[^ ^27^ ^]^ Raman spectroscopy (Figure 2b,c) strongly supported the high graphitization degree of LVGC: the intensity ratio of the D peak (≈1350 cm^−1^) to the G peak (≈1580 cm^−1^) was significantly reduced compared to the uncatalyzed sample (LVC), and distinct 2D peaks (≈2680 cm^−1^) emerged, indicating increased structural ordering and the presence of few or thin layers of graphite flakes.^[^ ^28^, ^29^, ^30^ ^]^ The XRD pattern (Figure 2d; Figure S3, Supporting Information) further confirmed this observation, showing a sharp graphite (002) diffraction peak at 26.48° in LVGC.^[^ ^31^ ^]^ According to the Mering–Maire formula, its graphitization degree reached 89.4%.^[^ ^32^, ^33^ ^]^ Combined with X‐ray photoelectron spectroscopy (XPS) analysis (Figure S6, Supporting Information), these results confirmed that Fe catalysis successfully converted low‐value biomass into precursors with highly ordered sp^2^ carbon structures.

The prepared LVGC exhibited significant interlayer stacking (Figure S7, Supporting Information), characteristic of graphite, making it a suitable intermediate for preparing high‐performance conductive nanoflakes. To efficiently obtain LVGN appropriate for device fabrication, we adopted a scalable liquid‐phase mechanical exfoliation strategy assisted by cellulose nanofibers (CNF) (Figure S8, Supporting Information). This strategy innovatively introduced CNF as a multifunctional collaborative delamination and dispersion medium. Owing to its nanoscale dimensions and high aspect ratio (Figures S9 and S10, Supporting Information), CNF wedged into interlayer gaps under intense mechanical forces, reducing the energy barrier for delamination.^[^ ^34^, ^35^ ^]^ Simultaneously, the CNF network dispersed localized high stresses during ball milling and increased system viscosity, thereby enhancing shear forces and enabling the delamination of larger‐sized LVGNs.^[^ ^36^, ^37^ ^]^

Alternatively, due to the surface electronegativity characteristics of both CNF and LVGC (Figure S12, Supporting Information), CNF also serves as an efficient and green stabilizer during peeling, suppressing the reaggregation of peeled LVGNs through a dual mechanism of electrostatic repulsion and steric hindrance.^[^ ^38^, ^39^ ^]^ After systematic optimization of ball‐milling parameters (Figures S13 and S14, Supporting Information), atomic force microscopy (AFM) analysis (Figure 2e,f) confirmed the successful preparation of LVGN with a uniform thickness of ≈1.5 nm. HRTEM (Figure 2g) and TEM (Figure S15, Supporting Information) micrographs revealed few‐layer structures (mostly ≤ 5 layers) with a highly ordered honeycomb lattice. The corresponding selected area electron diffraction pattern also showed sharp, point‐like hexagonal lattice diffraction spots instead of polycrystalline rings, providing clear evidence of high crystallinity and few‐layer stacking (Figure 2h).^[^ ^40^ ^]^ The yield from the original LV to LVGN was calculated to be approximately (12.8 ± 3.35) % by weighing after drying and subtracting the weight of CNF.

Ultimately, the resulting LVGN/CNF dispersion exhibited excellent long‐term stability (Figures S16, Supporting Information). By coating it onto filter paper, the continuous conductivity of the LVGN/CNF coating was demonstrated through successfully lighting a bulb in a test circuit (Figure 2i). These results provide an ideal foundation for fabricating high‐performance sensors.

### 2.3 Structure of Dual‐Network Conductive Sponge

**Figure 3:**
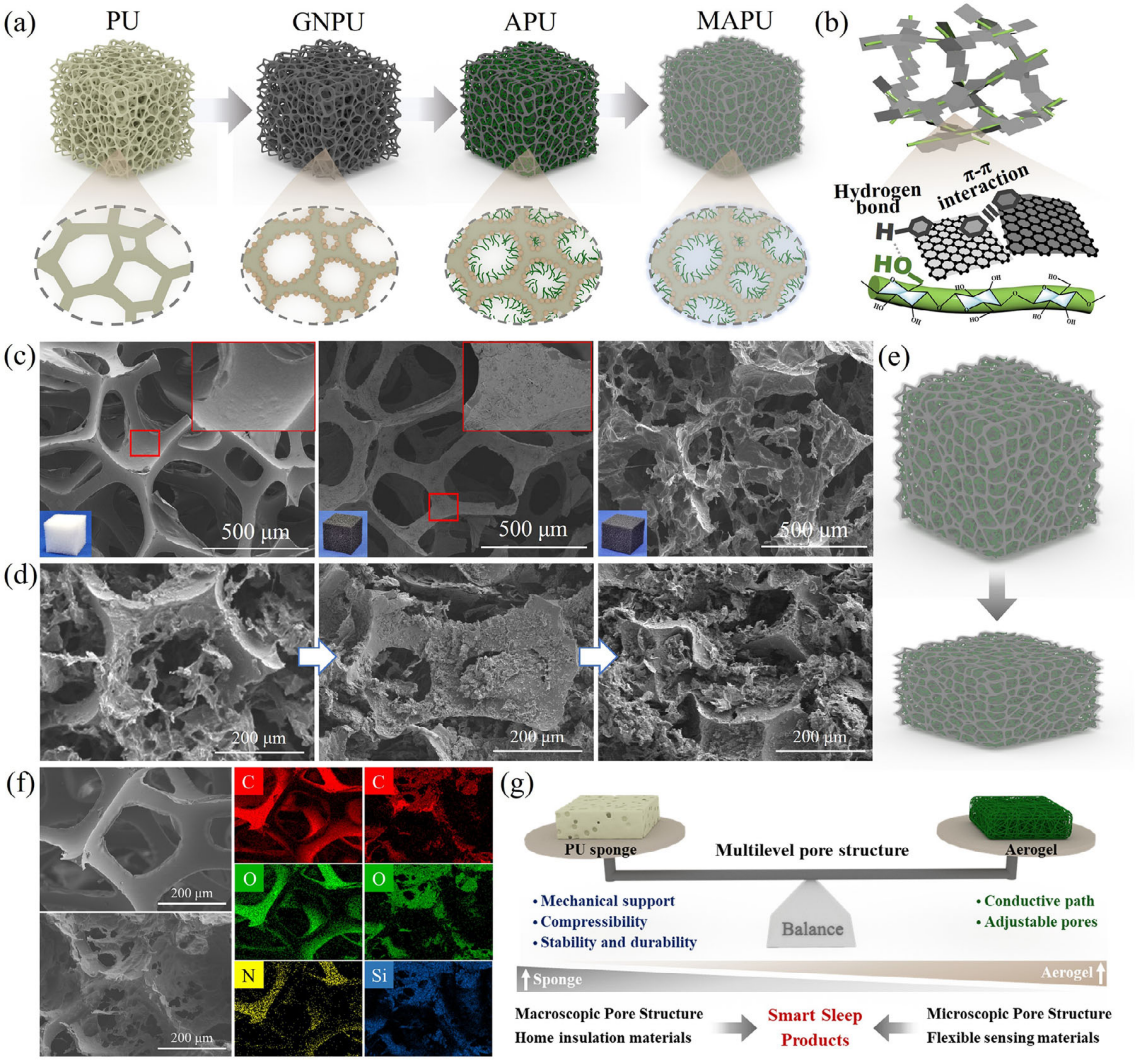
a) Schematic of sample structure at different preparation stages; b) molecular composition of dual‐network skeleton; c) SEM micrographs of MAPU at different preparation stages; d) SEM micrographs and e) schematic of MAPU undergoing compression; f) EDS images of PU and MAPU; g) role and balance of dual‐network skeleton.

The excellent performance of the MAPU conductive sponge originated from the multiscale synergy achieved through the multilevel fabrication strategy (**Figure** **3**a). First, the PU sponge was impregnated with prepared LVGN/CNF dispersion to construct a continuous and complete conductive framework (Figure 3c). During this process, by precisely controlling the number of coating cycles (Figure S17), the conductive layer fully covered the sponge structure while avoiding interfacial failure caused by excessive agglomeration. This provided a solid adhesive substrate for the growth of subsequent structures.

Based on this conductive framework, an in situ LVGN/CNF aerogel network was formed through a freeze‐induced self‐assembly technique. This mesoscopic sensing network provided a high specific surface area and sensitivity to small stresses within the macroscopic pores of PU through *π–π* stacking and hydrogen bond cross‐linking (Figure 3b; Figure S18, Supporting Information).^[^ ^41^, ^42^ ^]^ The resulting dual‐network heterostructure combined the macroscopic PU sponge skeleton for mechanical support with the mesoscopic conductive network for sensing functionality. Finally, the entire dual‐network structure was structurally fixed and interfacially enhanced through PDMS infiltration and solidification, imparting the device with excellent mechanical robustness. The introduction of a hydrophobic Si–O interface also endowed it with high stability in complex environments (Figure 3f; Figure S19, Supporting Information).^[^ ^43^ ^]^

The influence of PU sponge density and aerogel precursor concentration was investigated (Figure S21, Supporting Information). Larger pores provided greater deformation space and accommodated more conductive material, but significantly reduced the elastic modulus of the conductive sponge. Increasing the aerogel precursor concentration led to a higher aerogel network density, while causing a slight increase in the elastic modulus (Figure S22, Supporting Information). These findings demonstrate the universality of this multiscale conductive sponge fabrication strategy for biomimetic design, enabling sponges with tunable mechanical properties for diverse application requirements. Furthermore, to ensure consistent performance across batches of the same specification, we implemented a weight‐based screening protocol at each fabrication step (Figure S23, Supporting Information). This allows precise control over the incorporation amounts of different materials and the resulting multi‐level network structure, thereby achieving reproducible production (Figure S24, Supporting Information).

During compression, MAPU exhibited excellent resilience and structural stability due to the hierarchical force transmission and self‐protection mechanisms of the multilevel structure (Figure 3e). SEM analysis (Figure 3d) revealed that, in the initial compression stage, the PU skeleton deformed, rapidly reducing pore volume without completely closing it. The aerogel network pores remained open due to the separation between sponge walls. As the load increased, the sponge pores collapsed further until the pore walls contacted each other, leading to uniform transfer of the external force toward the interior. The aerogel network then cooperatively deformed, with its 3D skeleton elastically buckling while maintaining structural continuity. At maximum compression, both sponge pores and the aerogel network densified simultaneously to complete compaction. Because the sponge pore wall separates the aerogel network, it plays an effective role in stress dispersion, inhibiting crack growth and structural damage, providing the MAPU with superior robustness and durability.

The structural advantage of this multiscale design lies in the synergistic enhancement of mechanical and sensing performance at different scales. The in situ conductive gel network forms an interpenetrating reinforcement structure. The PU sponge, functioning as a macroscopic flexible support skeleton, provides excellent comfort and the ability to undergo large‐scale reversible deformation. The conductive layer attached to the skeleton establishes a stable conductive pathway, offers a growth interface for the conductive gel, builds strong physical entanglement and chemical bonding between hierarchical networks, and ensures effective stress transmission and efficient electron conduction. The finite element results reveal that the conductive aerogel network not only imparts the material with high sensitivity to minor deformations but also provides localized stress dispersion and secondary support mechanisms. This effectively suppresses the prolonged stress plateau typically observed during the macroscopic pore collapse and buckling of the PU foam, enabling the sensor to maintain highly linear responses across a broad stress range. The comparative analysis of compressive stress‐strain curves conclusively validates this mechanism (Figure S25, Supporting Information).

The final PDMS packaging process provides structural protection and serves as an environmental barrier, while also acting as a signal filter. Isolating conductive substances and suppressing contact slip prevents leakage currents and signal drift, significantly improving the signal‐to‐noise ratio and long‐term stability of the sensor.^[^ ^44^ ^]^ Overall, this strategy goes beyond simple material composites and provides a universal design approach for constructing flexible sensors that integrate comfort, high performance, and reliability.

### 2.4 Piezoresistive Properties of MAPU

**Figure 4:**
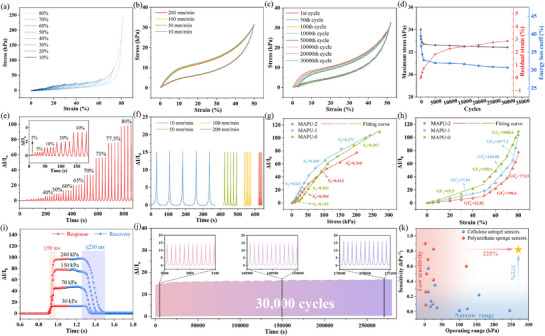
Compression recovery curves of MAPU‐1 under a) different strains and b) varying speeds; c, d) mechanical properties of MAPU under 30,000 compression cycles; current change rate of MAPU‐1 sensor e) under different strains and f) at different compression speeds; g) sensitivity and h) gauge factor of MAPU‐0, MAPU‐1, and MAPU‐2; i) response time of MAPU sensor during rapid loading and unloading at different pressure; j) current change rate curve of MAPU‐1 subjected to repeated loading and unloading at 50% strain for 30k cycles; k) sensitivity and operating range of MAPU compared with those of similar sensors recently reported in literature.

With its multiscale biomimetic structure, MAPU exhibited excellent piezoresistive properties, including long‐term mechanical durability and highly sensitive, stable sensing performance. Load–unload testing revealed its excellent energy dissipation and structural toughness (**Figure** **4**a,b; Figure S26, Supporting Information). The significant hysteresis loop observed during cyclic compression was due to the intrinsic viscoelasticity of the material and the gas viscosity resistance within the multistage pore structure.^[^ ^45^ ^]^ This is the physical basis for its use as a furniture cushioning material to provide comfort and energy dissipation.

In sharp contrast to traditional aerogel sensors, which are prone to structural fatigue failure due to their brittle framework, the ordinary single network sponge sensor benefits from the high toughness sponge matrix and can achieve 95.6% of the maximum stress retention rate and 1.56% of the residual strain under 10 000 times of cyclic compression with 50% strain (Figure S27, Supporting Information). MAPU foam also perfectly inherits the excellent mechanical properties of the PU sponge matrix. The highly tough PU sponge skeleton withstands and disperses most mechanical stress while separating and constraining the aerogel network. This design inhibited macro‐scale stress concentration and crack propagation. Therefore, while introducing an aerogel network, it still maintains excellent mechanical durability. After 30 000 cycles of high‐strain (50%) compression, the material largely retained its macroscopic structural integrity and core mechanical properties (Figure 4c). Specifically, its maximum stress retention rate reached 95.3%, while the residual strain was only 2.86%, fully demonstrating its enormous potential for long‐term service as a wearable device (Figure 4d). Notably, its energy dissipation rate stabilized from an initial 39.5% to 30.7%. This minor performance drift only occurred within the first few cycles, corresponding to the adaptive reconstruction of a small number of weak microstructures in the material, after which the material entered a highly stable cycling plateau.^[^ ^46^ ^]^ These results indicate that the multiscale biomimetic design successfully limited irreversible structural damage to microscopic and negligible local areas, thereby ensuring ultra‐high stability of overall macroscopic performance.

Multiscale biomimetic structures not only provide materials with excellent mechanical properties but also enable extraordinary piezoresistive sensing performance, converting macroscopic stress and strain into quantifiable electrical signals with high fidelity. We systematically evaluated the response characteristics of the MAPU sensor and found that, within an ultra‐wide strain range of up to 80%, its current signal exhibited a highly recognizable, monotonic correspondence with the applied pressure (Figure 4e). This response remained stable across different loading rates (Figure 4f), demonstrating its reliability in complex application scenarios. Both the response and recovery times increase with rising pressure, yet remain within the millisecond range within the operating range (≤50 and ≤250 ms, respectively) (Figure 4i), enabling accurate capture of high‐frequency dynamic events such as human movement. As a control, a sponge sensor without a multi‐level network structure also exhibits a response/recovery speed in the order of milliseconds (Figure S28, Supporting Information). This reflects the contribution of the excellent compressive resilience of the sponge matrix to this performance.

More importantly, the core advantage of the multiscale biomimetic structure lies in its ability to adjust key sensor performance indicators, particularly sensor sensitivity and operating range, by matching the density of different hierarchical networks. Results showed that increasing the network density of the aerogel effectively reduces the sheet resistance of the composite material and improves the strain sensitivity of the sensor (Figure 4h). However, an excessively dense aerogel network reduces material porosity, potentially leading to pore blockage and compromised compressibility, thereby diminishing stress sensitivity (Figure 4g; Figure S22, Supporting Information). Therefore, it is essential to optimize the aerogel content to achieve improved mechanical and electrical properties while preserving the open porous structure and excellent compressibility of the PU matrix. Among them, MAPU‐1 achieved a high sensitivity of 0.821 kPa^−1^ in the 0–53 kPa range, while maintaining considerable sensitivity values of 0.45 and 0.27 kPa^−1^ in the two higher pressure intervals of 50–100 kPa and 100–242 kPa, respectively. The increase in sponge density significantly expanded the stress response range of the sensor (Figure S22, Supporting Information). The medium‐density sponge enabled the sensor to achieve a wide measurement range (up to 242 kPa) while maintaining excellent sensitivity, offering broader application potential. This on‐demand performance control capability surpassed traditional single‐component sensors. In contrast, constrained by the stress plateau effect, the sponge foam sensor without a multi‐scale network structure exhibits a limited pressure response range of only 0–30 kPa and demonstrates markedly inferior sensitivity linearity (Figure S28, Supporting Information). This comparison underscores the substantial enhancement in sensing performance achieved by the multi‐scale network architecture.

A more rigorous test involved evaluating signal stability under long‐term cycling. After 30 000 cycles of high strain (50%), the response signal fluctuation of the sensor was successfully suppressed to 17.5% (Figure 4j). In contrast, the signal fluctuation of the sponge sensor without an aerogel network reached 27.4% after only 2000 cycles due to the weak interface binding force (Figure S27, Supporting Information). This once again demonstrates the crucial role of the separation‐limitation mechanism in protecting the internal conductive network. Compared with similar advanced sensors reported in recent literature (Figure 4k; Table S1, Supporting Information), MAPU demonstrated clear advantages, simultaneously achieving high sensitivity, a broad response range, and excellent cycling stability, three core indicators that are typically difficult to balance. This outcome highlights multiscale structural design as a highly promising approach to overcome the performance bottleneck of traditional flexible sensors.

### 2.5 Sensor Mechanism and Human Monitoring

**Figure 5:**
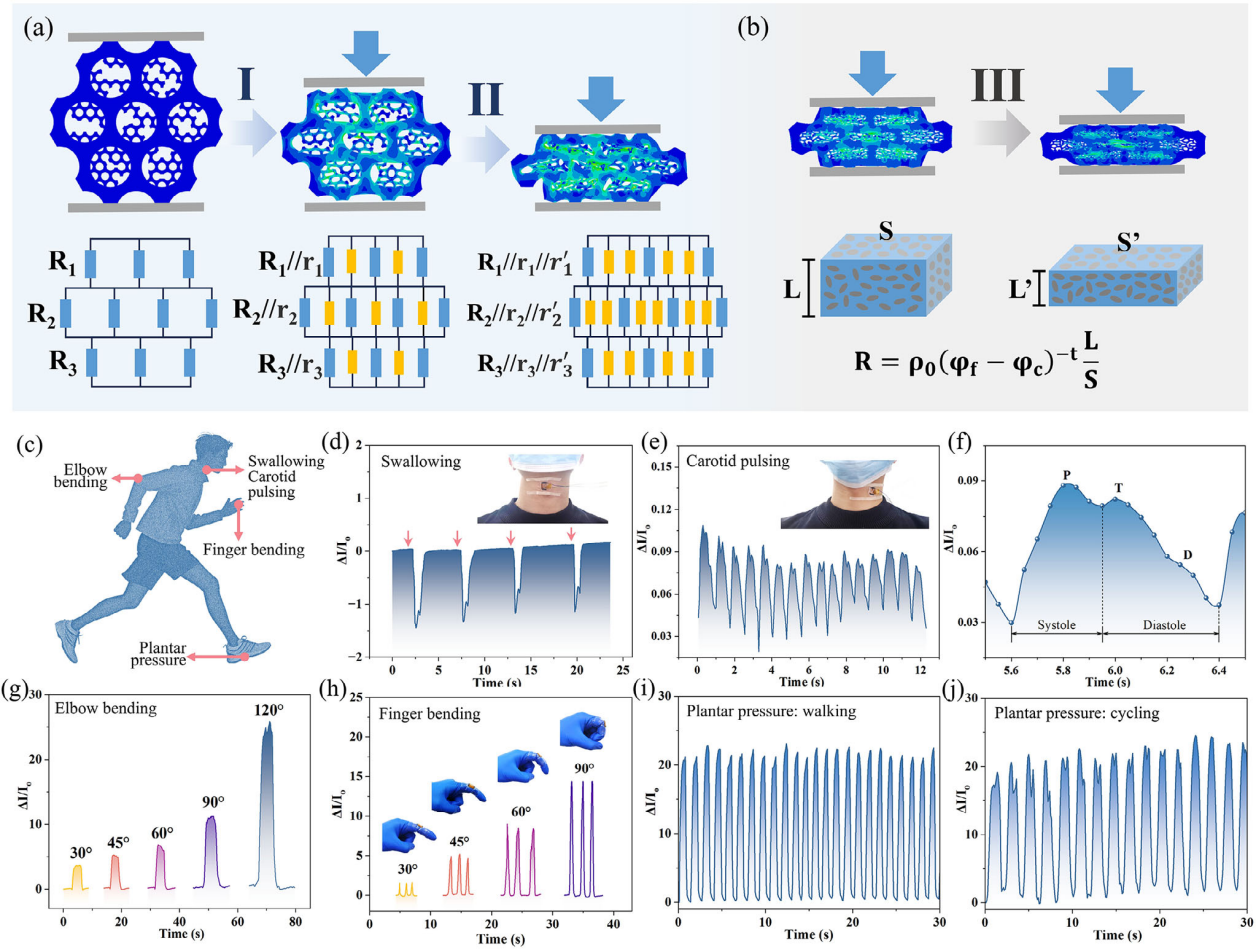
Pressure response mechanism of MAPU sensor during a) pore compression stage and b) densification stage; c) schematic of MAPU sensors monitoring various human motion signals; signal profiles for d) swallowing and e, f) carotid pulsing; detection of (g) elbow and (h) finger joint bending angles; detection of foot pressure during i) walking and j) cycling.

The exceptional sensing performance of the multiscale structure across a wide pressure range stems from two distinct response mechanisms operating in different pressure ranges. At low and medium pressures (Phases I and II), the response was governed by contact resistance changes (**Figure** **5**a). In this regime, MAPU functioned as a staged‐activation conductive network. Specifically, during initial loading (Phase I), minimal applied stress first induced elastic deformation of the flexible PU skeleton. This slight structural distortion initiated contact between adjacent aerogel conductive network units coated on the skeleton framework, thereby activating primary conductive pathways that were highly sensitive to minute pressure stimuli. As pressure progressively increased (Phase II), sequential collapse of sponge pores promoted extensive contact formation across a broader conductive network, specifically between the conductive layer on the PU skeleton surface and the aerogel network distributed throughout the pore architecture. Physically, this corresponded to a rapid increase in parallel conductive pathways within the resistive network, resulting in a sharp decrease in the total resistance of the circuit (Figure 5a).^[^ ^47^, ^48^ ^]^

In striking contrast, the control sample without aerogel infusion exhibited fundamentally constrained performance due to the absence of a high‐surface‐area 3D conductive architecture. During the initial elastic deformation phase, the conductive pathways of the control sample remained largely unchanged, and at higher compressions, the change in conductivity relied solely on limited contact points along the 2D skeleton surface (Figure S31, Supporting Information). This structural limitation severely compromised pressure detection capability across a broad range. Therefore, the staged activation mechanism enabled by the multiscale architecture provided the physical basis for achieving ultrahigh sensitivity in both low‐ and medium‐pressure regimes.

As structural densification began (Phase III) and contact resistance contributions approached saturation, a piezoresistive mechanism governed by percolation theory was seamlessly activated, thereby maintaining high sensitivity in the high‐pressure regime. Under extreme compression, the internal aerogel network collapsed into a nearly pore‐free solid, triggering two primary physical transformations. First, following percolation theory, the volume fraction (φ) of conductive phases (LVGN) substantially increased within the compressed matrix, effectively reducing the intrinsic resistivity of the material (Equation (7), Supporting Information).^[^ ^49^ ^]^ Second, macroscopic geometric effects caused the length of the sensor (L) to decrease while its cross‐sectional area (S) expanded due to the positive Poisson's ratio effect, collectively driving down the overall resistance (R = ρL/S) (Figure 5b). This intrinsic piezoresistive mechanism was essentially absent in control samples lacking compressible conductive aerogel filler, resulting in flattened high‐pressure response profiles. Therefore, the seamless transition and coordination between contact‐ and percolation‐dominated mechanisms collectively enabled the MAPU sensor to simultaneously achieve both high sensitivity and a broad detection range, surpassing the fundamental performance limitations inherent to traditional flexible sensors.

Systematic structural optimization and a multimechanism coupling design provided the MAPU sensor with high‐sensitivity pressure response across broad thresholds, demonstrating exceptional potential for physiological monitoring (Figure 5c). Leveraging the synergistic dual‐response mechanisms, the MAPU sensor achieved precise detection of subtle physiological signals such as swallowing motions and carotid artery pulses (Figure 5d,e). This advanced capability enabled real‐time pulse frequency monitoring with millisecond precision and facilitated accurate identification of characteristic arterial waveforms, including percussion (P), tidal (T), and diastolic (D) waves (Figure 5f).^[^ ^50^ ^]^ These demonstrate its strong potential for cardiovascular health monitoring and early disease warning systems.

Beyond vital sign monitoring, the MAPU sensor demonstrated superior flexibility and mechanical robustness in tracking both elbow and finger joint flexion (Figure 5g,h), providing a reliable platform for wearable biosensing and robotic motion capture applications. Furthermore, when embedded in smart insoles, the system achieved continuous real‐time monitoring of plantar pressure and movement (Figure S32, Supporting Information). During dynamic activities such as walking and cycling (Figure 5i,j), it demonstrated exceptional compressive responsiveness, a wide detection range, and rapid response and recovery. Collectively, these demonstrations establish MAPU sensors as a versatile platform for diverse applications in health monitoring, assistive medical devices, and intelligent robotic systems, providing a solid foundation for engineering applications of flexible sensing materials.

### 2.6 Functional Characterization of MAPU

**Figure 6:**
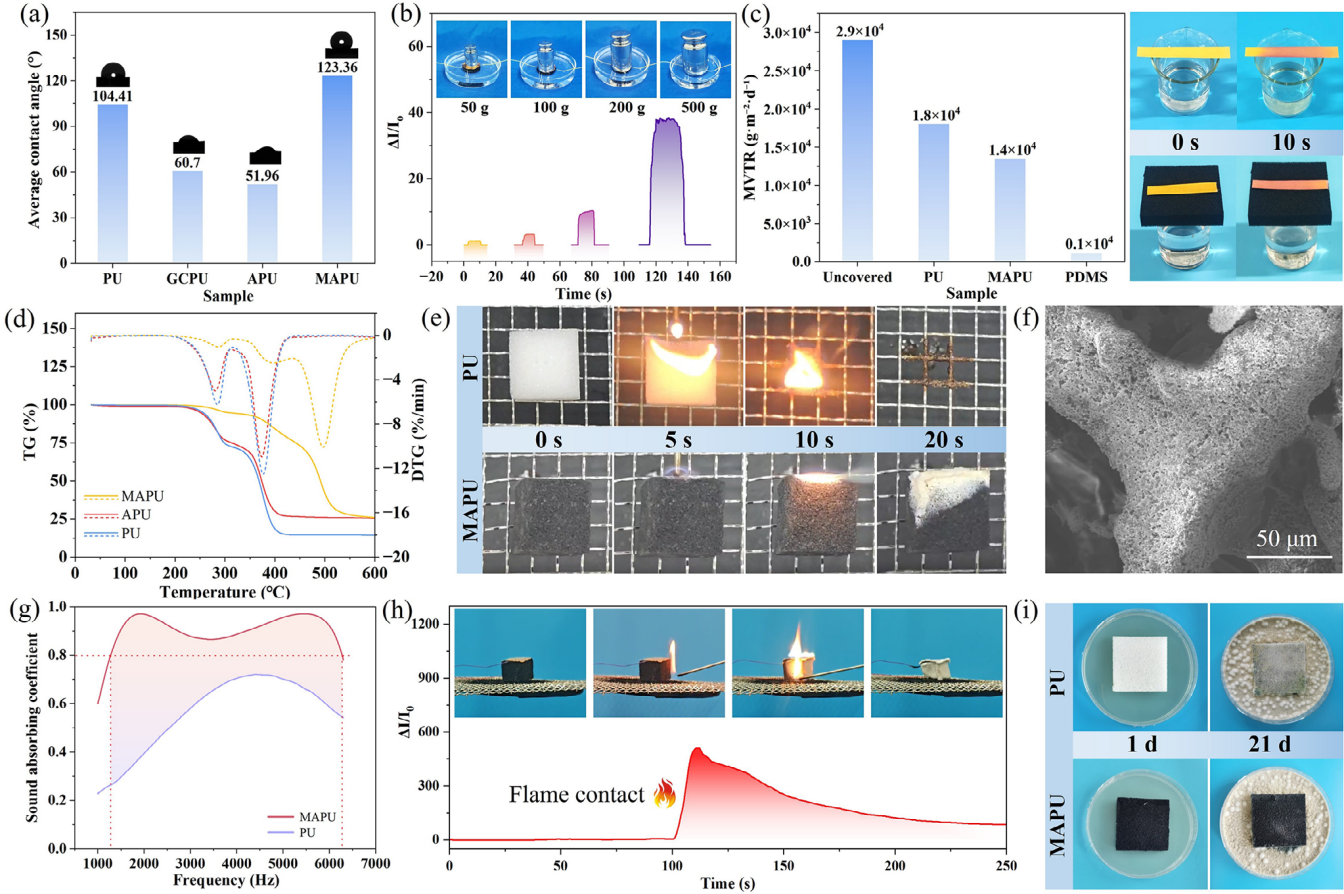
a) Water contact angle of samples at different preparation stages; b) signal detection in water; c) water vapor transmission rate of MAPU compared with PDMS film, PU sponge, and uncovered sample; d) TG and DTG curves of MAPU, APU, and PU sponge; e) comparison of flammability between MAPU and PU sponge; f) SEM micrograph of MAPU after combustion; g) sound absorption performance of MAPU compared with PU sponge; h) signal change of MAPU sensor during fire contact; i) antimold performance of MAPU compared with PU sponge.

This study presents a multiscale fabrication strategy that achieves breakthrough sensor performance while addressing compatibility challenges between electronics and the human body. The strategy enables the creation of truly human‐centric functional materials. For practical applications, especially in smart bedding requiring prolonged skin contact, seamless integration and thermal‐wet comfort are critical. MAPU exhibited superior design in these aspects. A PDMS coating provided exceptional surface hydrophobicity (**Figure** **6**a), forming a robust physical barrier for the internal conductive network.^[^ ^51^ ^]^ This barrier prevented liquid water penetration, eliminating performance drift or failure in humid environments (Figure S33, Supporting Information) and enabling underwater operation and wash durability (Figure 6b).^[^ ^44^, ^52^ ^]^

Additionally, while acting as an effective barrier to liquid water, the multilevel porous structure facilitated high‐throughput transport of gaseous water molecules.^[^ ^53^ ^]^ With a water vapor transmission rate of 1.4 × 10⁴ g·m^−2^·d^−1^ and air transmittance of 68.84 L·m^−2^·s^−1^, MAPU far exceeded the physiological requirements of human skin (200−500 g·m^−2^·d^−1^) (Figure 6c; Figure S34, Supporting Information).^[^ ^54^ ^]^ This enables excellent thermal‐wet management, preventing discomfort from humidity buildup while maintaining user comfort and health. The multilevel porous structure served as a channel for thermal‐wet management while also forming complex sound wave propagation paths. Sound waves strike the pore wall, converting sound energy into thermal energy through viscous resistance and damping.^[^ ^55^ ^]^ Through acoustic impedance matching across different pore scales, it achieved superior broadband noise absorption (1300–6300 Hz) with coefficients exceeding 0.8, creating a tranquil resting environment (Figure 6g; Figure S35, Supporting Information).^[^ ^56^ ^]^

MAPU represents an innovative integration of advanced sensing and home furnishing functions, requiring comprehensive comfort and safety considerations. Conventional furniture cushioning materials are highly flammable and frequently cause fire accidents. In contrast, the PDMS coating and LVGN conductive layer provide the MAPU material with a dual protective barrier, significantly enhancing its fire safety. The PDMS coating decomposed at high temperatures to form a dense SiO_2_ insulation layer while releasing noncombustible gases such as H_2_O and CO_2_ (Figure 6e; Figure S36, Supporting Information).^[^ ^57^ ^]^ The LVGN layer facilitates heat dissipation and promotes char formation, encouraging polymer cross‐linking into a more stable and compact carbonized layer that further enhances the barrier effect. Benefiting from this synergistic mechanism, MAPU exhibits significantly improved thermal stability compared to conventional PU foam. Horizontal burning tests confirm that MAPU achieves an HBF‐grade flame‐retardant rating (Table S2, Supporting Information). Its Limiting Oxygen Index (LOI) reaches 23.5%, substantially higher than the 16.3% of ordinary PU foam, while the heat release rate and total heat release are also markedly reduced (Figure S37, Supporting Information). Crucially, MAPU maintained its structural integrity and conductive pathways even after flame exposure and partial carbonization (Figure 6f). The carbonization of the skeleton caused a sudden drop in overall resistance, and the current signal exhibited a characteristic step that far exceeded the normal operating threshold, transforming the sensor from a pressure‐responsive device into a fire alarm sensor and enabling active fire prevention (Figure 6h). Moreover, the hydrophobicity and bioinertness of the PDMS coating effectively inhibited microbial growth, reducing the mold growth grade from Grade 4 to 2 (Figure 6i; Figure S38 and Table S3, Supporting Information). This enables biocompatibility and hygiene persistence under continuous human contact.

In summary, these multidimensional comfort and safety features are not isolated functional combinations but manifestations of an integrated design concept. They demonstrate the deep coupling and collaborative optimization of high‐performance sensing, passive protection, and active warning functions at the material‐constituent level, enabling the development of safe and comfortable intelligent wearable and home devices.

### 2.7 Application of MAPU Sensors in Intelligent Cushioning Materials

**Figure 7:**
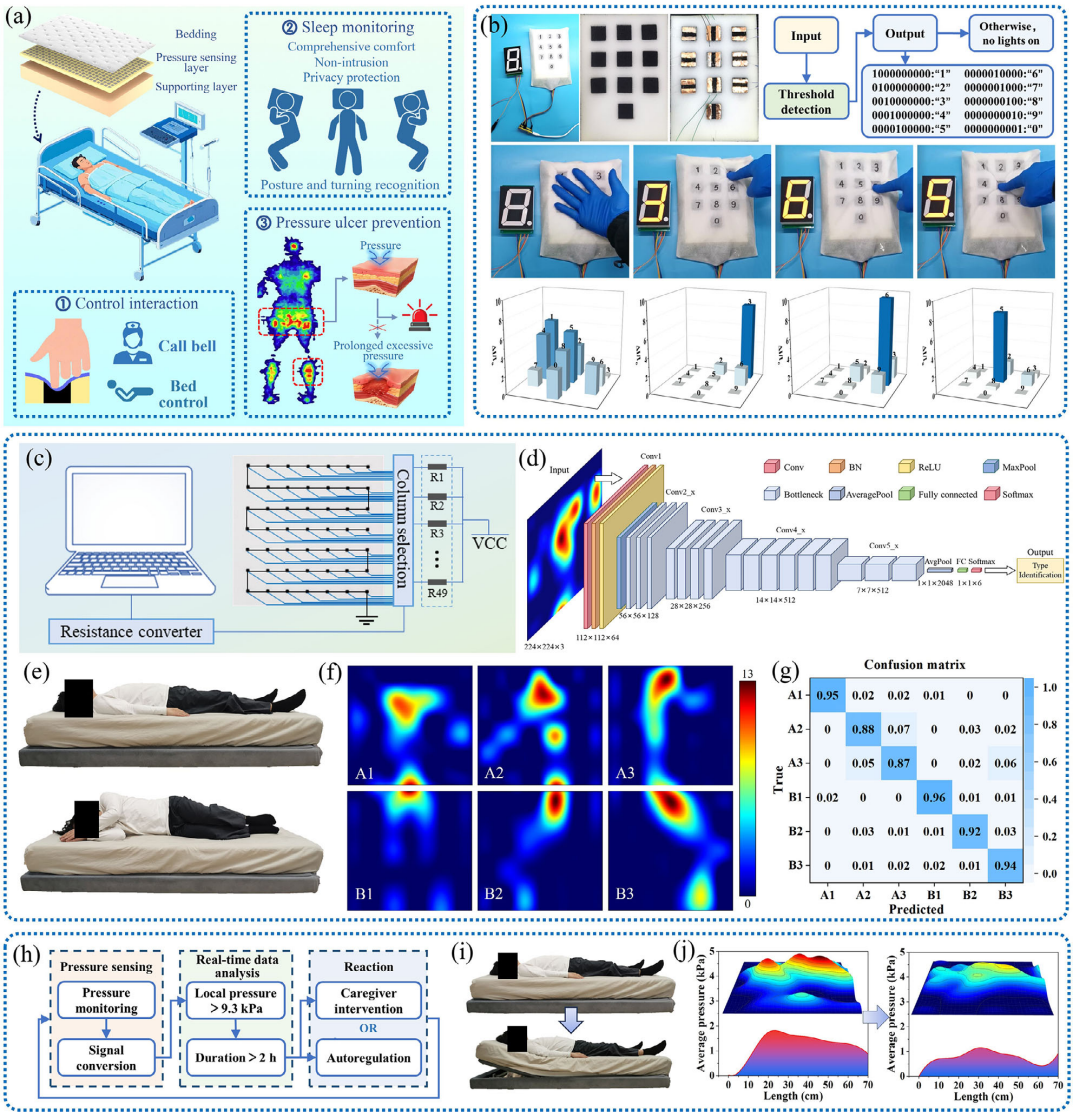
a) Application of MAPU sensors in a smart mattress; b) flexible buttons in a mattress sponge; c) connection diagram of the flexible sensor array; d) training flowchart of the network model; e, f) sleeping positions and corresponding body pressure distributions; g) confusion matrix produced by the trained model for real‐time evaluation; h) schematic of the pressure ulcer prevention system; i) bed angle adjustments and j) corresponding body pressure distributions.

Through systematic structural innovation and functional integration, MAPU has broken the barriers between electronic devices and flexible applications, demonstrating outstanding potential for smart furniture, particularly smart beds. Traditional film‐ or fabric‐based sensors used in current smart beds disrupt the human–bed interface continuity and create thermal‐wet barriers that compromise sleep comfort. In contrast, MAPU is a functional cushioning material. It can provide mechanical support, along with thermal and moisture management, while also implementing embedded pressure‐sensing functions, thereby enabling undisturbed sleep monitoring. This makes it a suitable material for applications in personalized health management and clinical pressure ulcer prevention (**Figure** **7**a).

In addition to passive monitoring, the excellent flexibility and elasticity of MAPU also make it suitable for flexible human–machine interaction interfaces. In this application, traditional rigid buttons can be replaced by seamlessly integrating interactive functions into a flexible substrate. By setting independent response thresholds for each sensing unit, a flexible interactive array composed of MAPU sensors was demonstrated to effectively suppress crosstalk and false touches, while also accurately interpreting complex spatial pressure inputs (Figure 7b). This showcases the vast potential of MAPU in flexible interactive interfaces, indicating the possibility of deeper integration among everyday flexible items, such as furniture, clothing, and intelligent devices.

To verify the comprehensive performance of the MAPU‐integrated pressure‐sensing system in practical use, this study thoroughly investigated the potential of MAPU sensor array‐based smart mattresses for body pressure distribution monitoring, sleep posture recognition, and pressure ulcer prevention. By embedding the MAPU sensor unit into the mattress sponge layer, a 7 × 7 sensor array was constructed (Figure 7c; Figure S39, Supporting Information). The sensor array converted spatial pressure distribution into electrical signals, transmitted them to the processing system, and presented the smoothed human pressure distribution map in real time through a graphical user interface (GUI) (Figure S40, Supporting Information). The body pressure distributions of adult subjects in supine, left lateral, and right lateral positions were obtained through video recording, providing a reliable tool for the non‐intrusive assessment of sleep movement and position. Using a convolutional neural network (VGG architecture) (Figure 7d), pressure heatmap samples were trained and tested to achieve intelligent classification of two zones (upper body and legs) and three postures (supine, left lateral, and right lateral) of the human body (Figure 7e,f).

The loss curves during training demonstrate favorable convergence behavior without significant overfitting or underfitting. The clustering visualization reveals that samples corresponding to different sleep postures and body regions form distinct clusters in the feature space, indicating that our sensor array effectively captures characteristic differences among various sleeping postures and body parts, thereby laying a solid foundation for subsequent posture classification (Figure S41, Supporting Information). The confusion matrix generated by the test showed an average recognition accuracy of up to 92% (Figure 7g), verifying the technical feasibility of the system in intelligent physiological recognition. Considering the potential influence of different factors on recognition performance, we categorized test samples by BMI body type and gender, and generated corresponding confusion matrices for analysis (Figures S42 and S43, Supporting Information). Here, a BMI less than 18.5 is classified as underweight, 18.5–24 as average weight, and greater than 24 as overweight. The model achieved an average recognition accuracy of 89% for underweight postures, which is lower than the 93% for average weight and 94% for overweight postures. This may be attributed to the limited number of sensing points in the sensor array, which might not fully capture the body pressure distribution characteristics of underweight individuals. Additionally, the model's average recognition accuracy for female subjects was 91%, lower than the 93% for males. This difference may be due to the higher proportion of underweight individuals among the female participants. These results confirm the wide applicability of the model to different users and underscore the practical advantages of the MAPU‐machine learning integration.

Furthermore, based on the pressure distribution of the mattress and the critical threshold for pressure ulcers (9.3 kPa/2 h),^[^ ^58^ ^]^ we designed a pressure‐ulcer‐prevention feedback system based on real‐time monitoring of body pressure (Figure 7h). The system automatically tracked local high‐pressure areas and calculated their durations, issuing timed turning prompts to caregivers or automatically adjusting the bed plate angle to optimize the body pressure distribution. The functional verification experiment showed that by lifting the bed board 30°, the average body pressure and maximum local pressure of the subjects were significantly reduced, alleviating the potential risk of pressure ulcers (Figure 7i,j; Figure S44, Supporting Information).

In summary, this work not only demonstrated the application of high‐performance flexible sensing materials in multichannel integration and complex human–bed interaction and monitoring, but also utilized material innovation to drive the development of an integrated health monitoring system combining sensing, decision‐making, and intervention. It pioneers a new technological pathway for intelligent, human‐centered preventive healthcare and comfortable living environments.

## 3 Conclusion

Addressing the urgent demand for flexible sensors in smart home and healthcare applications, this study presents a highly promising, low‐cost, and sustainable solution. We efficiently converted agricultural waste (LV) into biomass‐derived graphitic nanoflakes. Leveraging an innovative hierarchical assembly approach, we constructed a multifunctional conductive sponge (MAPU) using a PU sponge as the matrix. The core advantage of this material lies in the seamless integration of its multilevel conductive network and elastic skeleton, enabling outstanding sensing performance sufficient for human health monitoring and intelligent interaction applications (sensitivity: 0.821 kPa^−1^, response range: 242 kPa, stable response over >30 000 cycles).

Beyond focusing solely on core sensing performance, this study also prioritized practical deployment and real‐world functionality. Through deliberate material design, MAPU was endowed with multiple practical characteristics, including washability, flame retardancy, breathability, and sound absorption. This holistic approach enabled the development of a truly application‐oriented intelligent material. Smart mattresses equipped with these sensors successfully demonstrated real‐time recognition of human posture and intelligent early warning of pressure‐ulcer (bedsore) risk.

This study achieves a remarkable transformation of low‐value agricultural waste into high‐performance smart devices. Consequently, this study not only opens a novel pathway for the large‐scale, low‐cost fabrication of high‐performance flexible sensors but also holds significant potential to propel the deep integration of smart sensing technology into everyday homes. Ultimately, it aims to advance personalized health management and intelligent lifestyles to a new phase.

## 4 Experimental Section

### Materials

LV was produced in Lu'an, Anhui, China, and ground to a 100‐mesh powder. PU sponges with different densities were purchased from Yongjia Sponge Co., Ltd. Fe(NO_3_)_3_·9H_2_O, TEMPO, and NaBr were obtained from Shanghai Macklin Biochemical Co., Ltd. C_2_H_4_O_2_, HCl, and H_2_O_2_ (30%) were purchased from Xilong Science Co., Ltd. Na_2_CO_3_, NaHCO_3_, NaClO (6%–14%), and PDMS (Sylgrad 184) were supplied by Shanghai Aladdin Co., Ltd. Conductive silver paste was purchased from Osborn Co. The deionized (DI) water used in the study was produced by a Colton ultrapure water treatment system.

### Preparation of the LVGN Dispersion

Five grams of luffa vine (LV) powder was immersed in 300 mL of 1 wt.% Fe(NO_3_)_3_ solution under continuous stirring for 24 h and subsequently oven‐dried at 80 °C. The Fe loading capacity of the dried LV powder was determined to be 2.48 mmol g^−1^. The Fe‐loaded powder was then pyrolyzed in a tube furnace under N_2_ atmosphere at a heating rate of 5 °C min^−1^ up to 1000 °C and maintained for 3 h. After cooling, the obtained material was ground, acid‐washed with 1 M HCl, rinsed with DI water and ethanol until neutral, and dried at 105 °C to yield LVGC.

For the wet ball milling process, 1 g of LVGC was combined with 20 mL of 1 wt.% TEMPO‐oxidized cellulose nanofiber (LVCNF) dispersion in a milling jar containing zirconia beads (diameters = 1, 3, and 5 mm; mass ratio = 5:3:2). The bead‐to‐material volume ratio was maintained at 1:1. Planetary ball milling (YXQM‐2L, China) was conducted at 400 rpm for 9 h to obtain a homogeneous LVGN dispersion.

### Fabrication of MAPU

To fabricate the conductive composites, PU sponge was immersed in the LVGN dispersion and subjected to repeated compression–immersion cycles. Excess liquid was removed, and the sponge was oven‐dried at 50 °C to yield the conductive sponge (GNPU). Subsequently, GNPU was reimpregnated with conductive ink, flash‐frozen at −65 °C, and freeze‐dried to form the aerogel–sponge composite elastomer (APU). A PDMS solution was prepared by mixing Stock Solution A and Curing Agent B at a 10:1 mass ratio, diluted 10 fold with n‐hexane. The APU composites were then dip‐coated in this solution and thermally cured at 80 °C, yielding MAPU. To investigate the impact of aerogel loading density on sensing performance, sponge with a density of 30 kg·m^−3^ was selected, and the precursor solution for GNPU impregnation was divided into three batches diluted with deionized water at volumetric ratios of 1:0 (undiluted), 1:0.5, and 1:1. The resulting gradient samples were designated as MAPU‐0, MAPU‐1, and MAPU‐2, respectively. In addition, to investigate the impact of sponge pore size on sensing performance, a moderate concentration of precursor solution was selected, and the sponge with densities of 20, 30, and 40 kg·m^−3^ was used as the matrix to prepare MAPU. The obtained samples were designated as MAPU‐20, MAPU‐30, and MAPU‐40, respectively.

### Assembly of the Piezoresistive Sensor and Sensor Array

For sensor fabrication, MAPU material was sectioned into 1.5 cm cubes. Opposing faces of each cube were coated with conductive silver paste, and copper foil electrodes with integrated copper wire leads were attached, forming simple piezoresistive sensing units. To integrate the array into the mattress, the standard 1.5‐cm‐thick sponge layer beneath the outermost fabric layer was modified by creating a 7 × 7 grid of cavities with 10 cm center‐to‐center spacing. The fabricated MAPU sensors were subsequently embedded into these cavities, forming the sensor array illustrated in the Figure S39 (Supporting Information).

### Characterization and Measurement

Detailed descriptions of the characterization methods and tests were provided in the Supporting Information. This study was approved by the Anhui Agricultural University Ethics Committee on Publishing and Scientific Research (No. KJLL2025047), and written informed consent was obtained from all participants.

## Conflict of Interest

The authors declare no conflict of interest.

## Supporting information



Supporting Information

Supplemental Video 1

Supplemental Video 2

Supplemental Video 3

Supplemental Video 4

Supplemental Video 5

Supplemental Video 6

## Data Availability

The data that support the findings of this study are available from the corresponding author upon reasonable request.
