# Supplementary material for: Biomimetic Multiscale‐Structured Biomass Graphene/Polyurethane Sponge Composite for Flexible Pressure Sensors and Intelligent Cushioning Materials
Source: Adv Sci (Weinh). 2025 Nov 25;13(8):e17511. doi: 10.1002/advs.202517511 (PMC12884719; doi:10.1002/advs.202517511)
Supplement: Supplementary file 1 — Supporting Information [file ADVS-13-e17511-s001.docx]

**Supporting Information**

**Biomimetic multiscale-structured biomass graphene/polyurethane sponge composite for flexible pressure sensors and intelligent cushioning materials**

Runmin Xu ^a,b^, Pusen Cao ^a,b^, Tingting Zhang ^a,b^, Jie Wei ^a,b^, Yue Wang ^a^, Zhi Huang ^c^, Zhenghang Chen ^a,b^, Jianfeng Xi ^a,b^, Yong Guo ^a,b,*^, Yuxia Chen ^a,b,d,*^

^a^ College of Materials and Chemistry, Anhui Agricultural University, Hefei 230036, China

^b^ Anhui Healthy Sleep Home Furnishings Engineering Research Center, Hefei 230036, China

^c^ Anhui Le Fu Er Home Technology Co., Ltd., Hefei 230036, China

^d^ Key Laboratory of Wood and Bamboo Furniture, National Forestry and Grassland Administration, Changsha, Hunan, China

Email: fly828828@163.com; sheherose@163.com;

**Supporting information includes:**

Experimental section

Figure S1–S44

Tables S1–S3

References

Videos S1–S6

**Experimental section**

**Preparation of LVCNF**

LV powder was added to a 1:1 (v/v) mixture of acetic acid and hydrogen peroxide solution, with a solid-to-liquid ratio of 1: 20. The mixture was stirred at 85 °C to prepare LV fibers. A sodium carbonate-sodium bicarbonate buffer solution (pH = 10.5) was prepared. 1 g of LV fiber was dispersed in 100 mL of the buffer solution and stirred for 20 minutes to obtain a homogeneous dispersion. 0.016 g of TEMPO (2,2,6,6-tetramethylpiperidine-1-oxyl) and 0.1 g of sodium bromide (NaBr) were sequentially added to the dispersion and stirred until dissolved. 10 mL of sodium hypochlorite (NaClO) solution was added to initiate the oxidation reaction. After stirring for 3 hours, the oxidation reaction was terminated with ethanol. The oxidized fibers were filtered and thoroughly washed with deionized water until the filtrate reached a neutral pH of 7. The washed oxidized fibers were re-dispersed in deionized water to form a 1 wt% dispersion. This dispersion was then subjected to ultrasonication for 10 minutes using a cell disrupter to obtain a homogeneous suspension of LVCNF.

**Morphological and chemical characterization methods**

The microstructure of the material was characterized by scanning electron microscopy (SEM, Hitachi S-4800, Japan), transmission electron microscopy (TEM, JEOL JEM-2100F, Japan), high-resolution transmission electron microscopy (HRTEM, JEM-2100plus, Japan), and atomic force microscopy (AFM, Bruker Dimension ICON, Germany). SEM had the function of X-ray energy spectrum analysis (EDS). X-ray diffraction (XRD, Smartlab SE, Japan) analysis was conducted to observe the crystal phase characteristics of carbon samples, the scanning speed was 2 °/min, and the starting and ending angles were 5 °–90 °, respectively. The defects and crystal structure of carbon samples were measured by Raman spectroscopy (Raman, Horiba LabRAM HR Evolution), at the laser wavelength of 532 nm, the test wavenumber ranges from 50 to 4000 cm^−1^. Four hundred spectral data were collected from a square area of 50 μm × 50 μm using area scanning mode. X-ray photoelectron spectroscopy (XPS, Thermo Kalpha, America) was used to determine the distribution and content of elements. The vantage software was used for the deconvolution of the high-resolution XPS spectra. The surface functional groups of samples were qualitatively analyzed by Fourier transform infrared (FTIR) spectroscopy (Nicolet Is20, America) for qualitative analysis via KBr squashing method, the frequencies were between 400 and 4000 cm^−1^ with a resolution of 4 cm^−1^. The Zeta potential analyzer (DLS, Malvern Zetasizer Nano ZS90, UK) was used to analyze the Zeta potential of biomass conductive ink. The thermal stability of samples was assessed via Thermogravimetric Analysis (PerkinElmer STA 6000, America) at temperatures ranging from 30 to 600 °C, with a heating rate of 10 °C/min, all conducted under nitrogen atmosphere. The DTG curve was obtained by differentiation. The hydrophobicity of the samples was tested using a contact angle tester (Inno CA100D, China).

**Calculation method for graphitization degree**

Graphitization degree is measured using XRD method. It can be calculated based on the Mering–Maire equation^[1,2]^, as follows:

$G={(0.3440-d_{002})}/{\left( 0.3440-0.3354 \right)\times100\%}$ (1)

where $G$ (%) is the degree of graphitization of the natural graphites, 0.3440 is the interlayer spacing of fully non-graphitized carbon (nm) and 0.3354 is the interlayer spacing of an ideal graphite crystallite (nm). The interlayer spacing ($d_{002}$) can be obtained from the XRD patterns using the Bragg equation^[3]^, as follows:

$2d\sin\theta=n\lambda$ (2)

where $d$ is the interplanar spacing, $\theta$ is the angle between the incident X-ray and the corresponding crystal plane, $\lambda$ is the wavelength of the X-ray, and $n$ is the diffraction order.

**Sensor performance testing method**

The compressive tests and mechanical properties of MAPU sensors were measured by a universal test machine (REM-710, China) with a 500 N load cell. The stress-strain curves were plotted. Specifically, the cyclic compression tests were performed in a controlled indoor environment at 24 °C and 50% relative humidity. The compressive strain was fixed at 50%, with a loading/unloading speed of 100 mm·min⁻¹, corresponding to a cycling frequency of approximately 0.11 Hz. Each test involved more than 30,000 repeated cycles, confirming the excellent long-term mechanical and electrical durability of the sensor. The energy loss coeff ($\eta$) is defined as the proportion of externally applied work dissipated during a loading-unloading cycle. It is calculated as the ratio of the hysteresis loop area to the area under the loading curve^[4]^, which was calculated using Eq. (3), as follows:

$\eta={\oint\delta d\varepsilon}/{\int\delta_{L}d\varepsilon}\times100\%$ (3)

where $\varepsilon$, $\delta$ and $\delta_{L}$ are the cyclic compress strain, loading-unloading curve, and unloading curve, respectively.

The electrical signal of MAPU sensors was measured using an electrochemical workstation (CHI660E, China). The rate of current change was expressed in terms of Eq. (4):

${\Delta I}/{I_{0}}=(I-I_{0})/I_{0}$ (4)

where $I_{0}$ is the initial current without pressure and $I$ is the real-time current at different pressures.

To further elaborate the variation of the sensor sensitivity, ${\Delta I}/{I_{0}}$ was linearly fitted to different stresses and the sensitivity (S kPa^−1^) was used as an indicator of the stress sensing sensitivity at different pressure ranges^[5]^, which was calculated by using Eq. (5). A linear fit analysis of ${\Delta I}/{I_{0}}$ across different strains and used the gauge factor (GF) as a metric of strain sensitivity^[6]^, which was calculated using Eq. (6), as follows:

$S=\delta({\Delta I}/{I_{0}})/\delta P$ (5)

$GF={\Delta I}/{I_{0}}/\Delta\varepsilon$ (6)

where $\delta P$ is the stress change and $\Delta\varepsilon$ denotes the strain change.

**Percolation theory model**

Percolation theory describes the process by which conductive fillers dispersed in an insulating matrix form a conductive network. When the concentration of conductive fillers reaches a critical threshold, interconnected pathways emerge between the fillers, causing a sharp drop in the material's electrical resistance^[7]^. According to the theory of percolation, the relationship between the conductivity of the composite material and the content of the conductive filler can be expressed by Eq. (7), as follows:

$\sigma=\sigma_{0}\left( \varphi_{f}-\varphi_{c} \right)^{t}$ (7)

where $\sigma$ and $\sigma_{0}$ stand for the conductivity of the composite material and conductive filler, $\varphi_{f}$ is the content of conductive filler in composite material, $\varphi_{c}$ represents the percolation threshold, $t$ reveals the critical factor. Since resistivity is the reciprocal of conductivity, the resistivity of the composite material can be expressed by Eq. (8), as follows:

$\rho=\rho_{0}\left( \varphi_{f}-\varphi_{c} \right)^{-t}$ (8)

where $\rho$ and $\rho_{0}$ stand for the resistivity of the composite material and conductive filler.

**Finite element analysis (FEA)**

FEA was used to investigate the compressive behavior of the MAPU sensor structures using ABAQUS software. Structural models of different sensors were first built according to their actual measured parameters, then imported into the software and meshed. A series of loads (static and dynamic forces) were applied to the upper surface of the sensing layer model to examine the generated strains, whereas the lower surface was constrained.

**Moisture resistance and water vapor transmission rate (WVTR) test**

MAPU sensors were first conditioned in a desiccator for 24 hours. Sensor performance was performed using a universal test machine (REM-710, China) coupled with an electrochemical workstation (CHI660E, China). Subsequently, sensors were transferred to an environmental chamber maintained at ambient temperature with 85% relative humidity (RH) for 24 hours. The same test was performed immediately after removal. Comparative analysis evaluated humidity-induced performance variations.

WVTR was tested with a protocol according to the the American Society for Testing Materials (ASTM) E96^[8]^. The samples were placed at the top of a bottle (containing 10 mL DI water) and the periphery was sealed with paraffin tape. At last, they were kept at room temperature and weighed every 24 h. The WVTR is calculated using Eq. (9), as follows:

$WVTR=G/{tA}$ (9)

where $G$ is the loss of weight (g), $t$ is the period elapsed during the test (d), and $A$ is the area of the test region (m^2^).

**Thermal conductivity test**

The thermal conductivity of specimens was characterized following ISO 22007-2 protocols, utilizing a hotdisk (TPS2500S, Sweden). The test temperature is 25℃. The thermal conductivity ($\lambda$) is calculated using using Eq. (10), as follows:

$\lambda=P\cdot D_{\left( t \right)}/\left[ \pi^{3/2}\cdot r\cdot\Delta T_{\left( t \right)} \right]$ (10)

where $P$ is the constant probe input power (W), $r$ is the sensor radius (m), $\Delta T_{\left( t \right)}$ is the transient average temperature rise (K) at measurement time τ, and $D_{\left( t \right)}$ is the dimensionless function derived from the double-spiral probe's thermal response model.

**Acoustic absorption characterization**

The sound absorption coefficient of specimens was measured using an impedance tube system (SW422 & SW477, China) according to GB/T 18696.2-2002. A four-microphone impedance tube configuration was employed to measure normal incidence absorption coefficients across 1/3-octave bands. Two specimen types with 4 cm thickness but differing diameters (10 cm for low-frequency testing at 63–1600 Hz and 3 cm for high-frequency testing at 1000–6300 Hz) were acoustically characterized. For each specimen type, three replicates were measured with rotational repositioning (two orientations per specimen), yielding six replicate datasets per group, and the arithmetic mean of these replicates was reported as the final result.

**Anti-mildew test**

The anti-mildew properties of MAPU, PU sponge were evaluated in accordance with the national standard GB/T18261-2013. The fungal strains Aspergillus niger (AS3.315) and Chaetomium globosum (AS3.3601) used in this experiment were obtained from the China General Microbiological Culture Collection Center. A 10 mL volume of sterile water was added to the cultured fungal strains. Under aseptic conditions, spores were gently harvested from the surface of the fungal cultures using an inoculation loop to prepare a spore suspension. Test specimens were sectioned to dimensions of 45 mm × 45 mm × 3 mm and subsequently sterilized by UV irradiation. Nutrient salt agar medium was dispensed into sterilized Petri dishes. Following solidification, the specimens were aseptically positioned onto the surface of the solidified medium. The mixed spore suspension was uniformly applied to the specimen surfaces via spraying. The inoculated Petri dishes were sealed and incubated at 28 °C with 85% relative humidity for 21 days. Fungal colonization on the specimens was evaluated by determining the percentage surface coverage area. Specimens were rated according to the established standard (**Table S3**).

**Fire resistance test**

To observe the combustion behavior of the specimens, the sample was placed on a metal wire mesh. A butane torch was positioned near one end of the specimen to ignite its side surface, with the ignition time maintained for 10 seconds. The combustion phenomena of the sample were recorded. Furthermore, the Limiting Oxygen Index (LOI) was determined according to ASTM D2863-23. Specimens were cut into strips measuring 10 mm × 10 mm × 150 mm, vertically clamped within a test column, and ignited at the top using a butane flame within an atmosphere of controlled oxygen-nitrogen mixture. The oxygen concentration was gradually decreased until the burned length of the specimen was less than 5 cm. The oxygen concentration at this point was recorded as the LOI value. Ten specimens were tested for each sample type, and the average value was reported.

**Signal acquisition of human body pressure distribution**

The mattress used in the experiment measured 200 cm × 90 cm. A 7 × 7 sensor array was embedded within a layer of polyurethane foam beneath the fabric cover of the mattress. The adjacent sensors were spaced 10 cm apart. Depending on placement orientation, the array detected pressure from either the upper body or legs to train sleep posture and body-part recognition models. Twenty subjects (10 males, 10 females; aged 20–30 years; mean height 168.25 ± 6.59 cm; mean weight 61.25 ± 7.46 kg; mean BMI 21.54 ± 1.74 kg·m⁻²) were positioned with their upper body or legs over the sensor area, adopting standardized supine, left lateral decubitus, and right lateral decubitus postures. Pressure distribution heatmaps were stochastically captured during this process, yielding 30 heatmaps per subject per posture (15 for upper body and 15 for legs), totaling 1,800 heatmaps for neural network training and testing. The sensor-embedded mattress was further placed on an adjustable electric bed with segmented elevation functionality. Subjects laid supine on the mattress with their torso positioned over the sensor array area. The back section of the bed platform was then elevated and the changes in pressure distribution were recorded.

**Machine learning for sleeping position recognition**

Raw thermal images captured by the MAPU array were preprocessed to standardize the input dimensions (224 × 224 × 3) before being fed into the CNN. Through successive convolutional and pooling layers, the spatially localized thermal-pressure features were extracted and gradually condensed into discriminative representations. These features were flattened and passed to a fully connected layer, followed by a SoftMax classifier with an output dimension of six corresponding to the posture categories. Model optimization was performed using the cross-entropy loss function and stochastic gradient descent (SGD) optimizer, enabling robust convergence and minimizing classification error. This methodological addition provides clearer insight into the analytical foundation of the sleep posture recognition system.

**Real-time position recognition system**

The real-time pressure monitoring system was constructed by integrating MAPU sensors, an STM32F103C8T6 microcontroller, piezoresistive voltage divider circuits, and ADC modules. The signals obtained from sensors (acquired at 100 Hz) were processed in real-time using the trained machine-learning model hosted on a PC for position recognition. Recognition results are visualized via a custom-designed graphical user interface (GUI), displaying dynamic pressure distribution maps and sleep position classification.


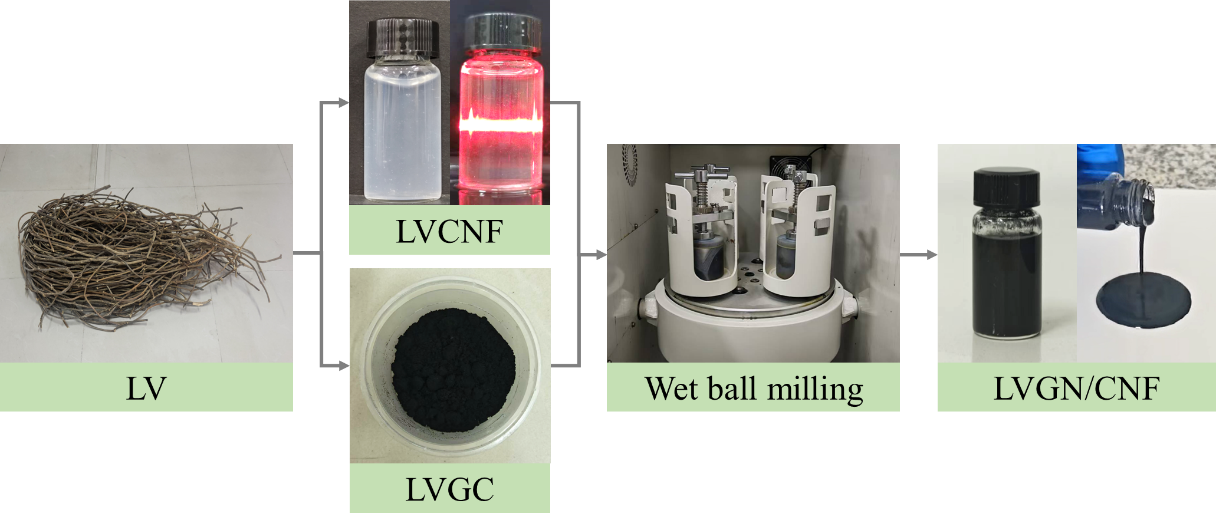


**Figure S1.** LVGN preparation process real object diagram.


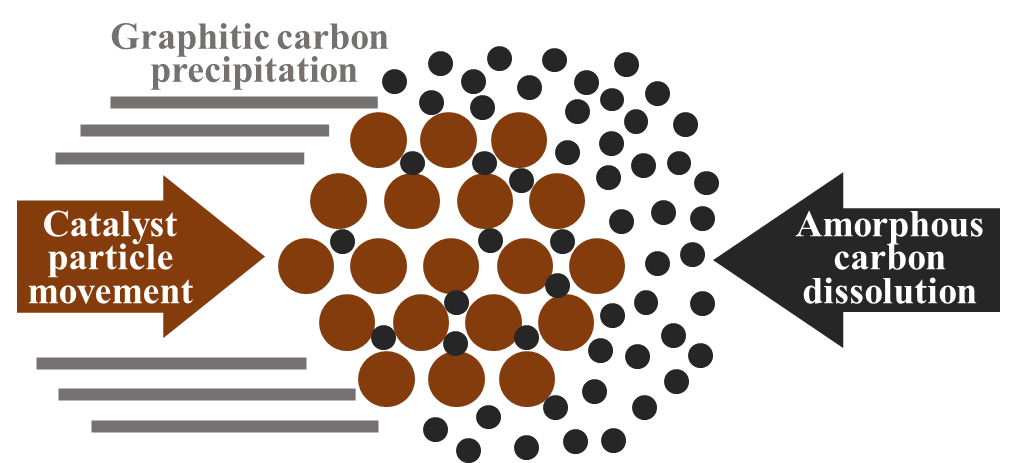


**Figure S2.** Schematic diagram of catalytic graphitization mechanism.


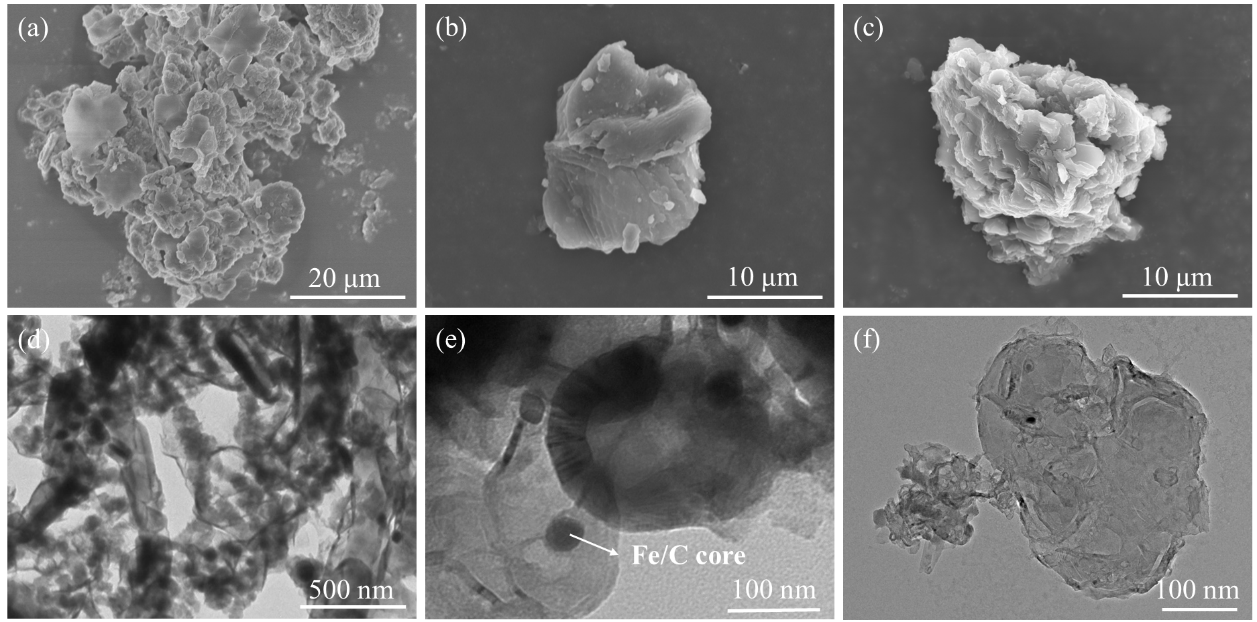


**Figure S3.** SEM micrographs of LVGC (a, b) before and (c) after acid washing; TEM micrographs of LVGC (d, e) before and (f) after acid washing.


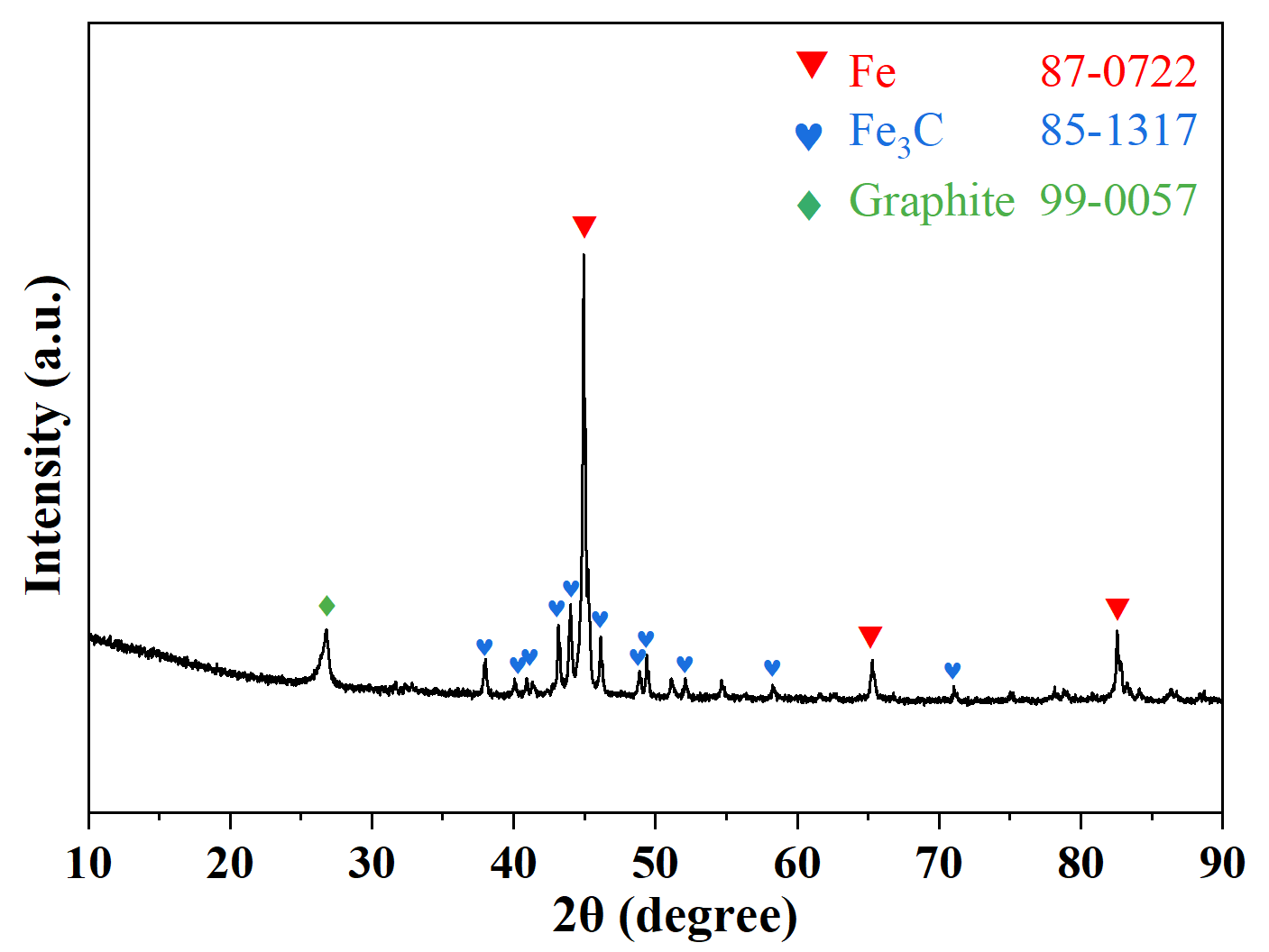


**Figure S4.** XRD pattern and phase analysis of LVGC before acid washing.


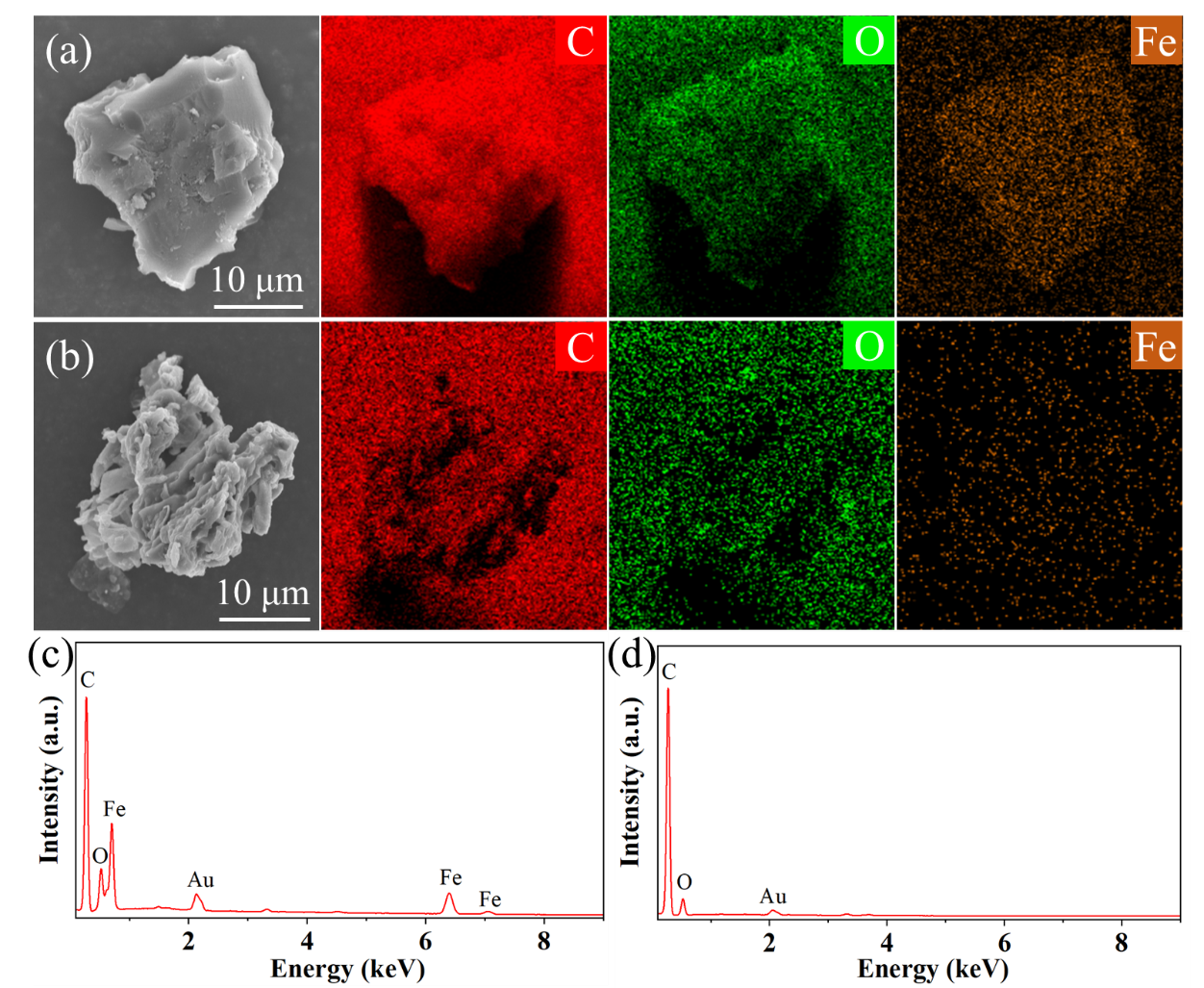


**Figure S5.** EDS images and element energy spectrum of LVGC (a, c) before and (b, d) after acid washing.


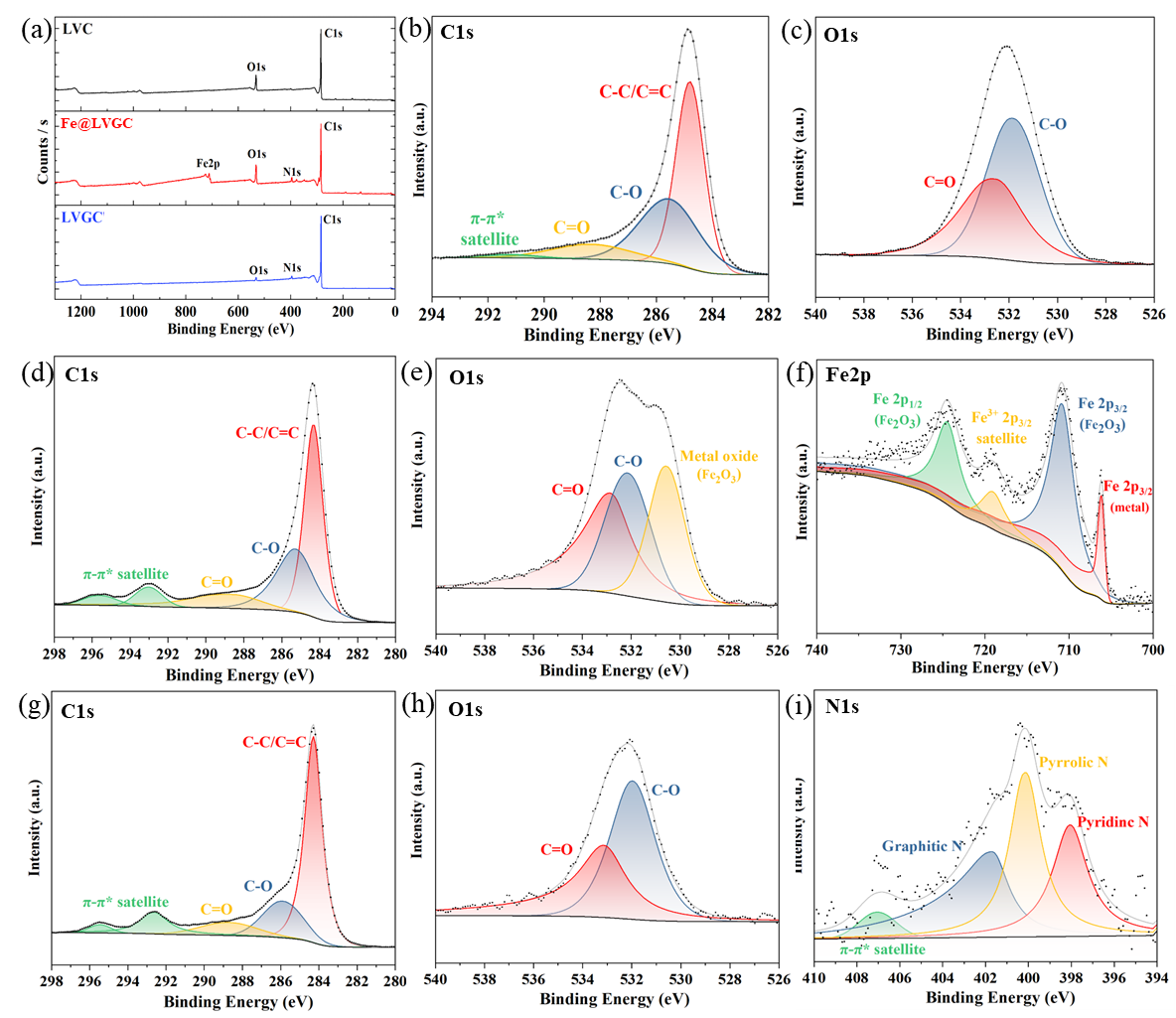


**Figure S6.** (a) XPS full spectrum of LVC, Fe@LVGC and LVGC; high-resolution (b) C spectrum, (c) O spectrum of LVC; high-resolution (d) C spectrum, (e) O spectrum, and (f) Fe spectrum of Fe@LVGC; high-resolution (g) C spectrum, (h) O spectrum, and (i) N spectrum of LVGC.


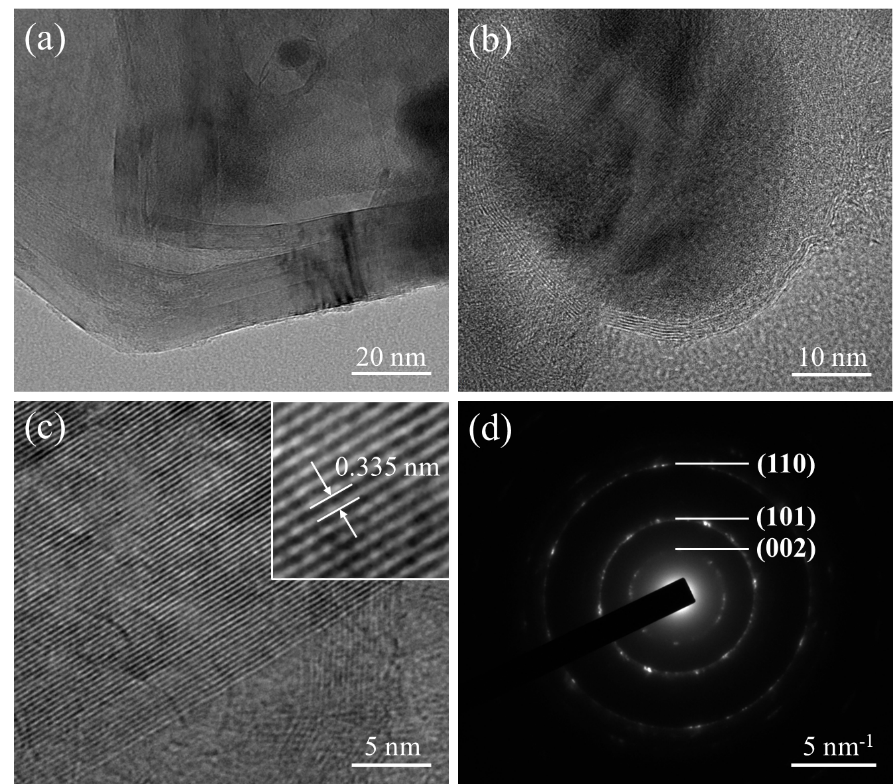


**Figure S7.** (a-c) HRTEM micrographs and (d) electron diffraction pattern of LVGC.


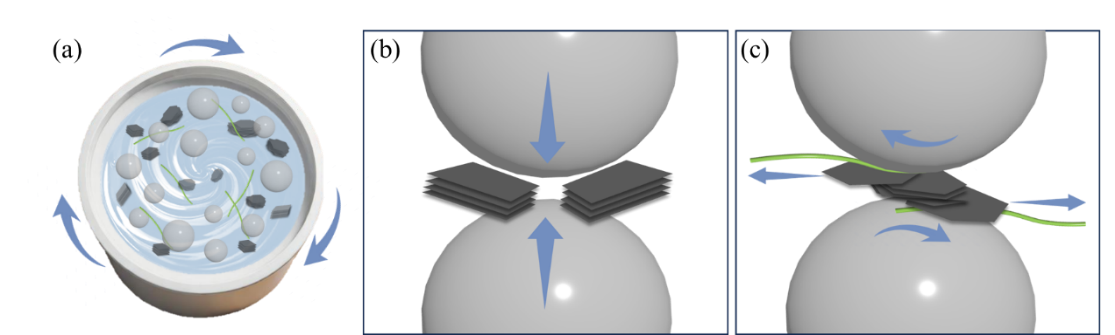


**Figure S8.** (a) Wet ball-milling, (b) fragmentation of graphite charcoal, and (c) exfoliation of graphene layers.


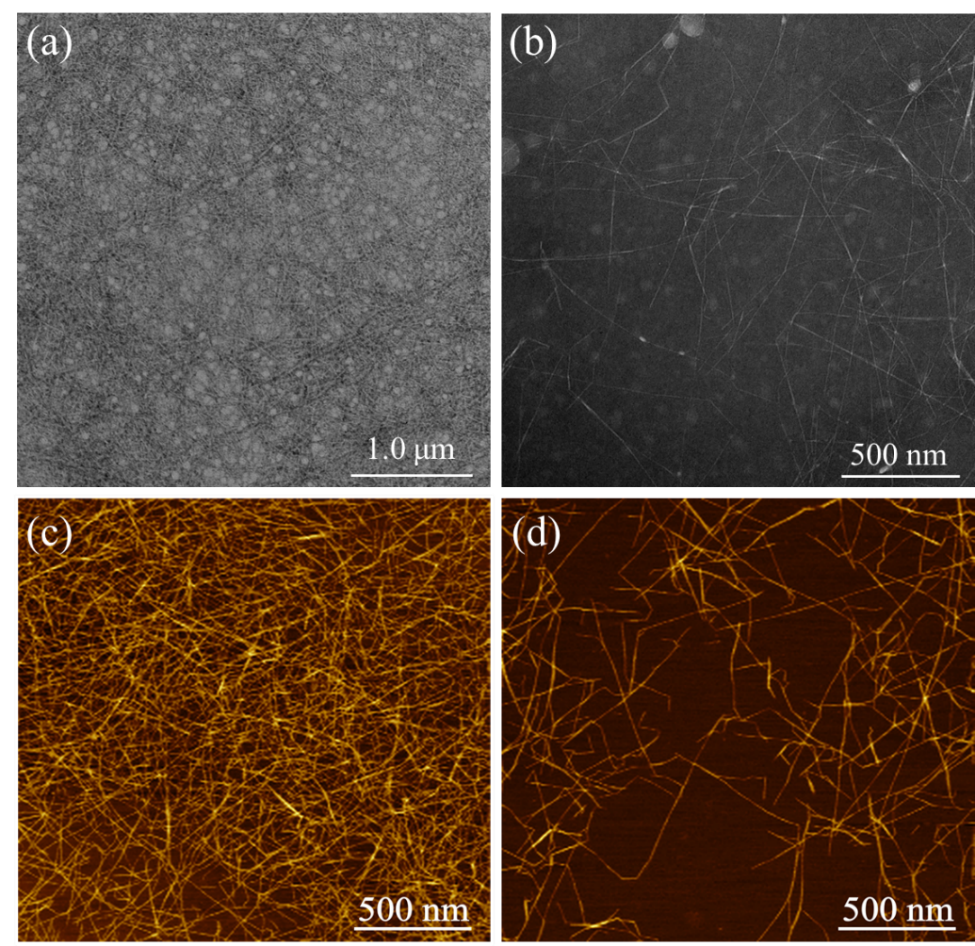


**Figure S9.** (a, b) TEM and (c, d) AFM micrographs of LVCNF dispersion and its diluted form.


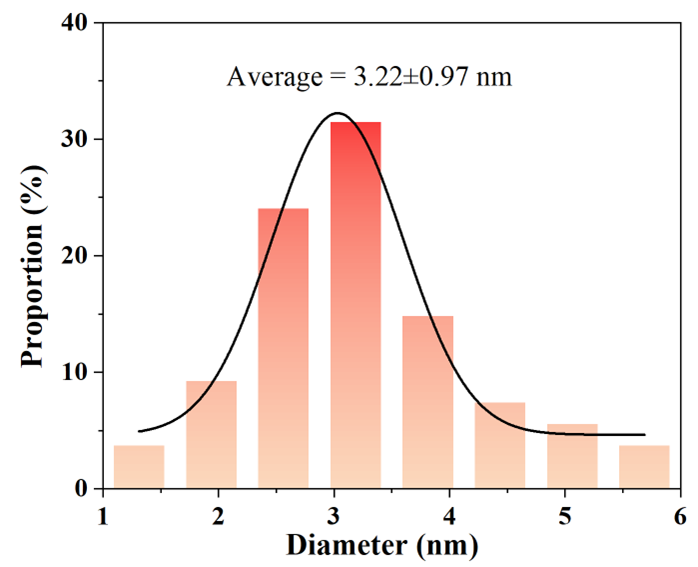


**Figure S10.** Diameter statistic of LVCNF.


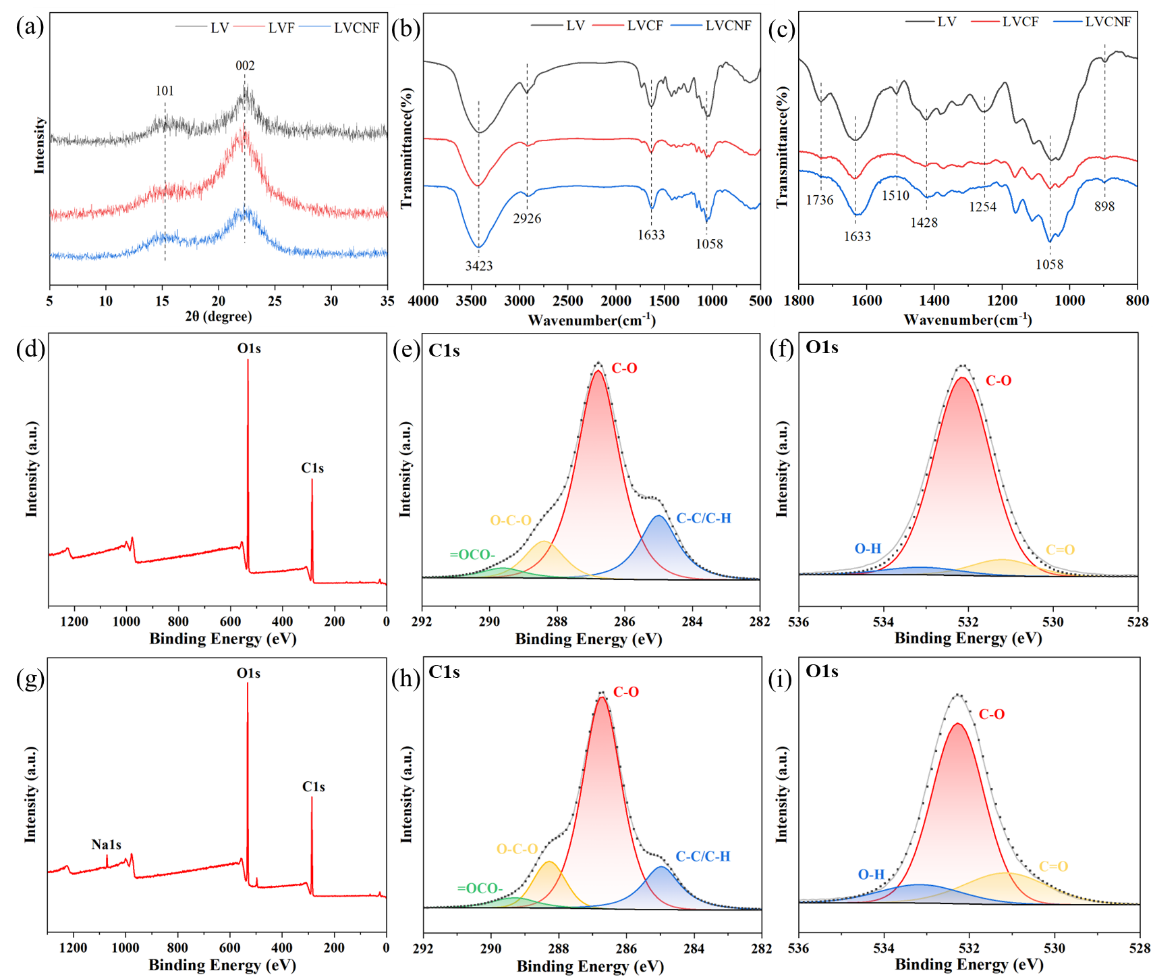


**Figure S11.** (a) X-ray diffraction patterns and (b, c) FTIR spectra of LV, LVF, and LVCNF; (d) XPS full spectra, high-resolution (e) C1s spectrum and (f) O1s spectrum of LVF; (g) XPS full spectra, high-resolution (h) C1s spectrum and (i) O1s spectrum of LVCNF.


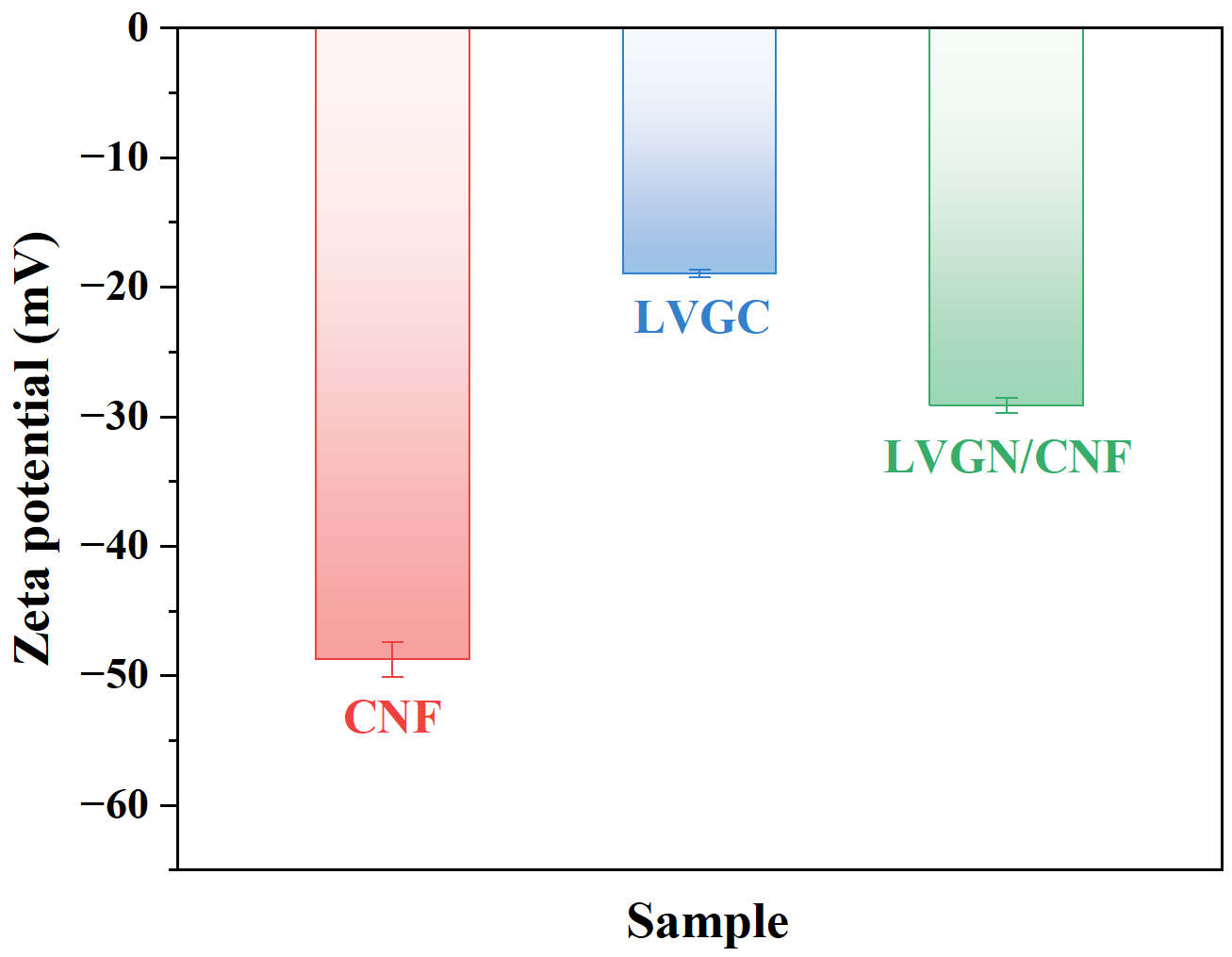


**Figure S12.** Zeta potential of CNF, LVGC and LVGN/CNF.


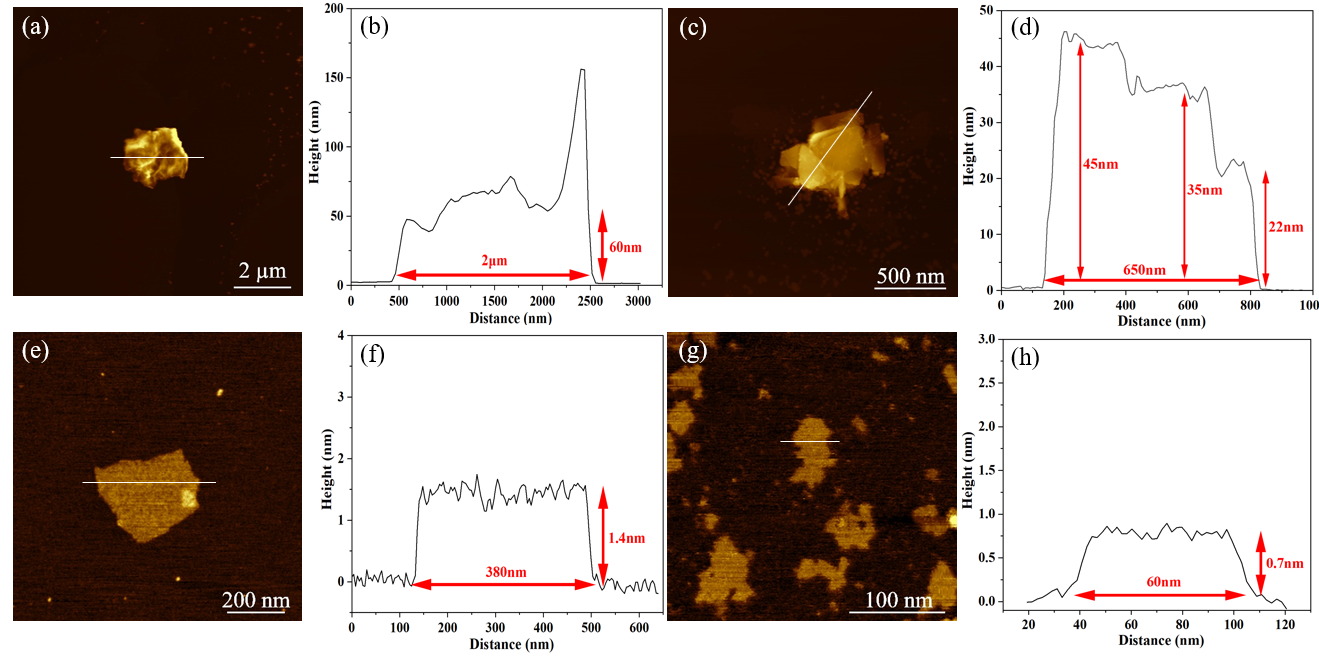


**Figure S13.** AFM micrographs and size measurement of LVGN obtained at different ball milling times: (a, b) 3 hours, (c, d) 6 hours, (e, f) 9 hours and (g, h) 12 hours.


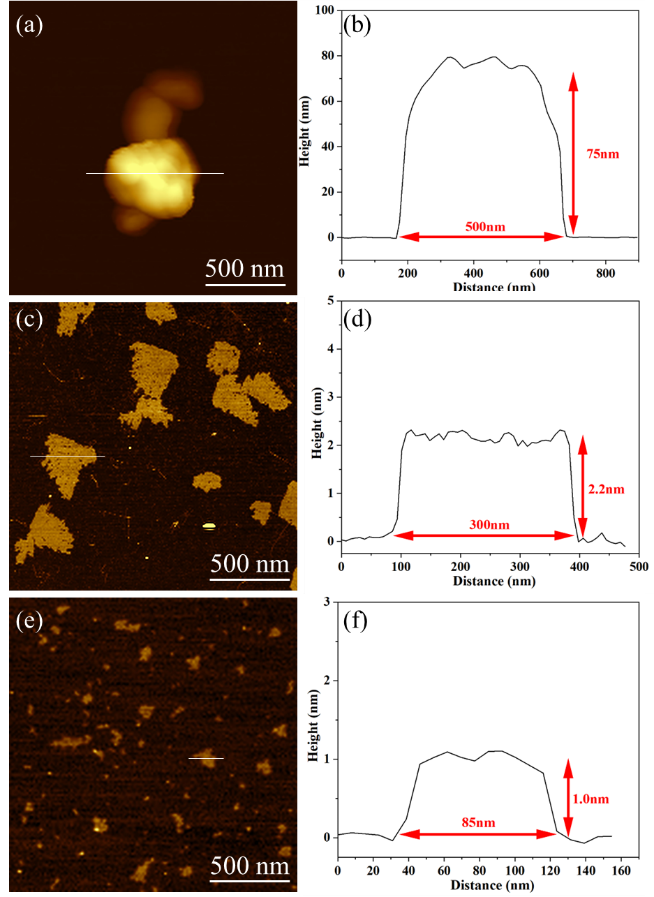


**Figure S14.** AFM micrographs and size measurement of LVGN at different ball milling speeds: (a, b) 200rpm, (c, d) 400rpm and (e, f) 600rpm.


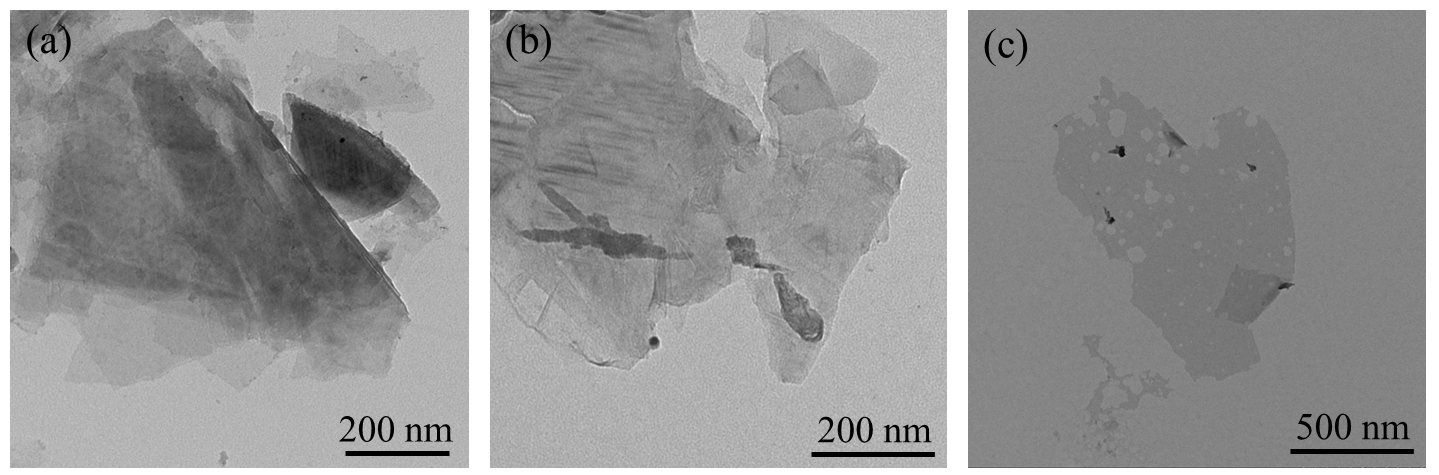


**Figure S15.** TEM micrographs of LVGN with few layers and single-layer graphene structures.


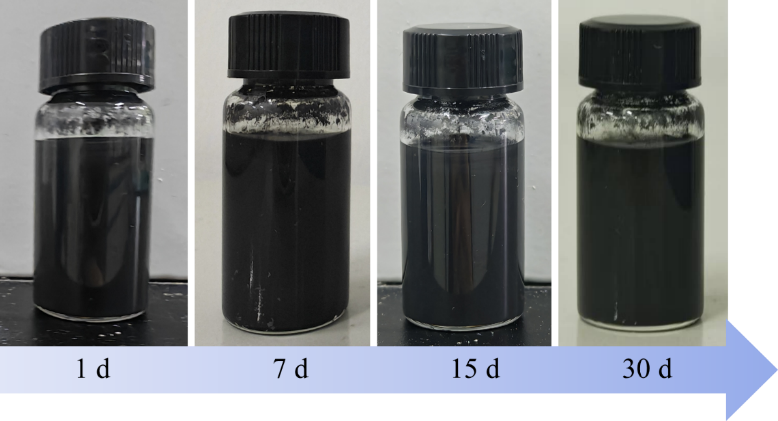


**Figure S16.** LVGN/CNF dispersion maintains excellent dispersion stability throughout 30-day static storage


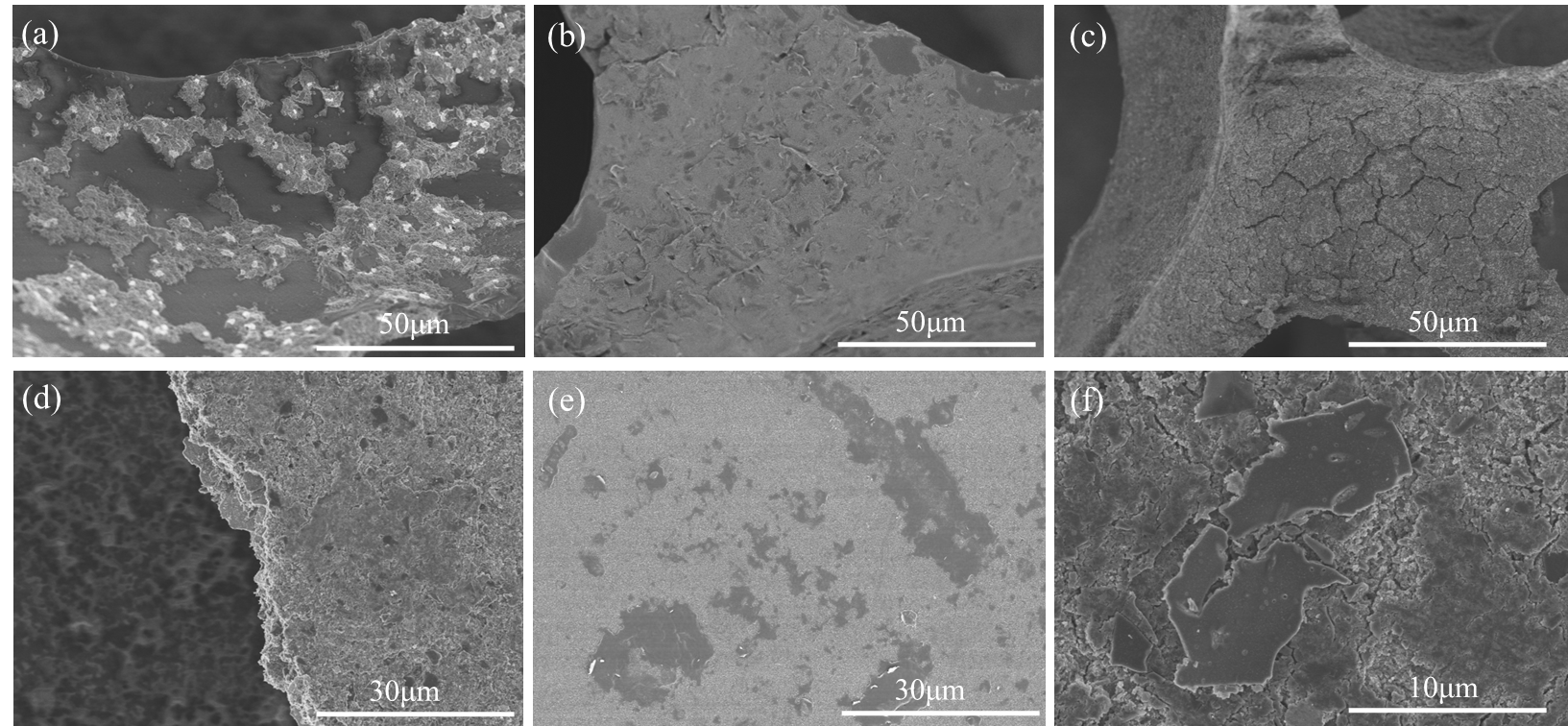


**Figure S17.** LVGN coating applied on PU with different coating times: (a) once, (b)twice, and (3) three times; (d-f) LVGN coating on PU surface.


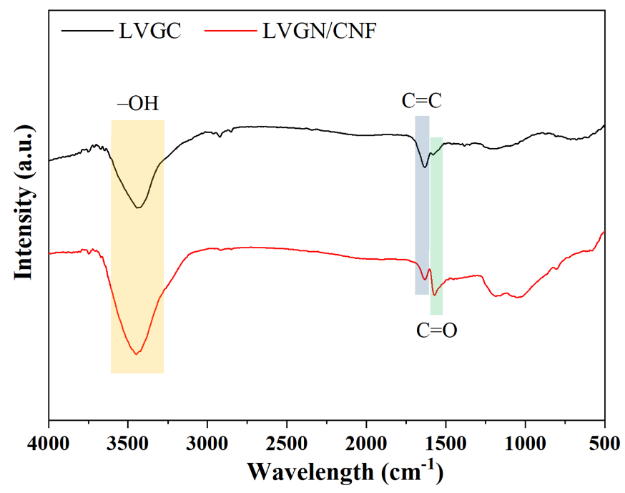


**Figure S18.** FTIR spectra of LVGN and LVGC/CNF.


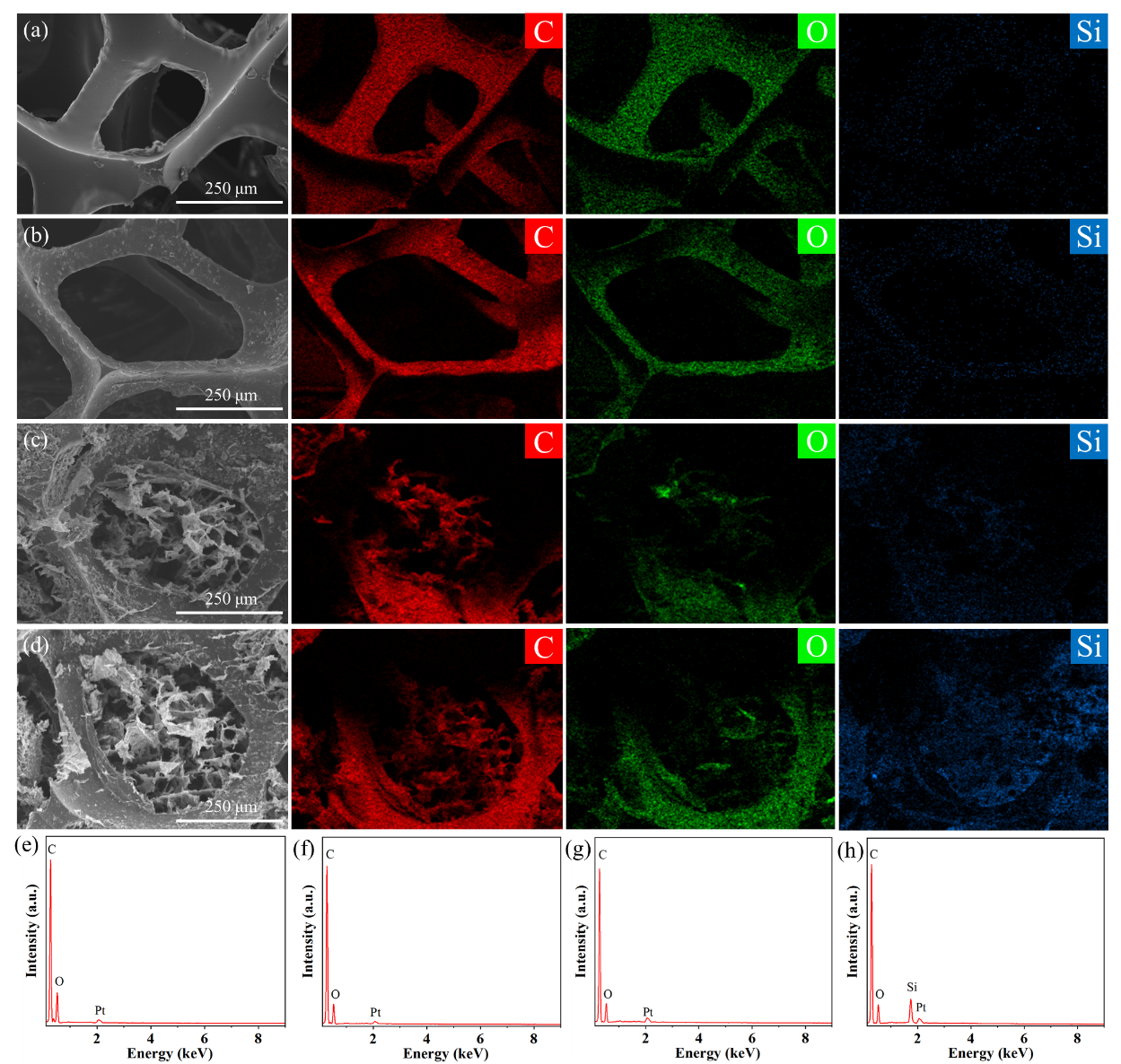


**Figure S19.** EDS images and element energy spectrum of MAPU at different preparation stages: (a, e) PU sponge, (b, f) GNPU, (c, g) APU and (d, h) MAPU


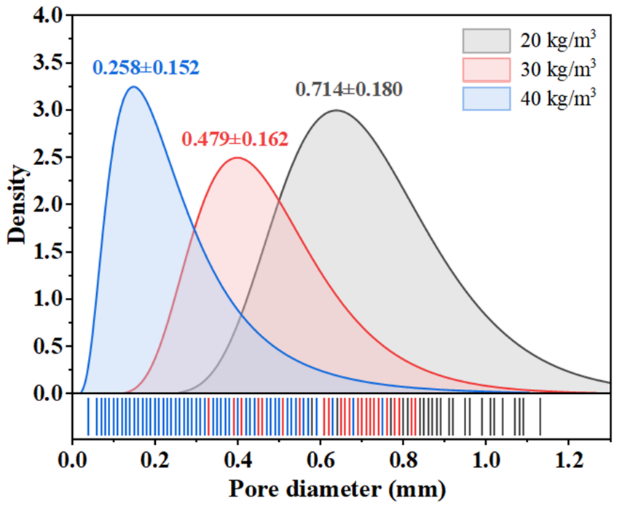


**Figure S20.** Pore size distribution of PU sponge with different densities.


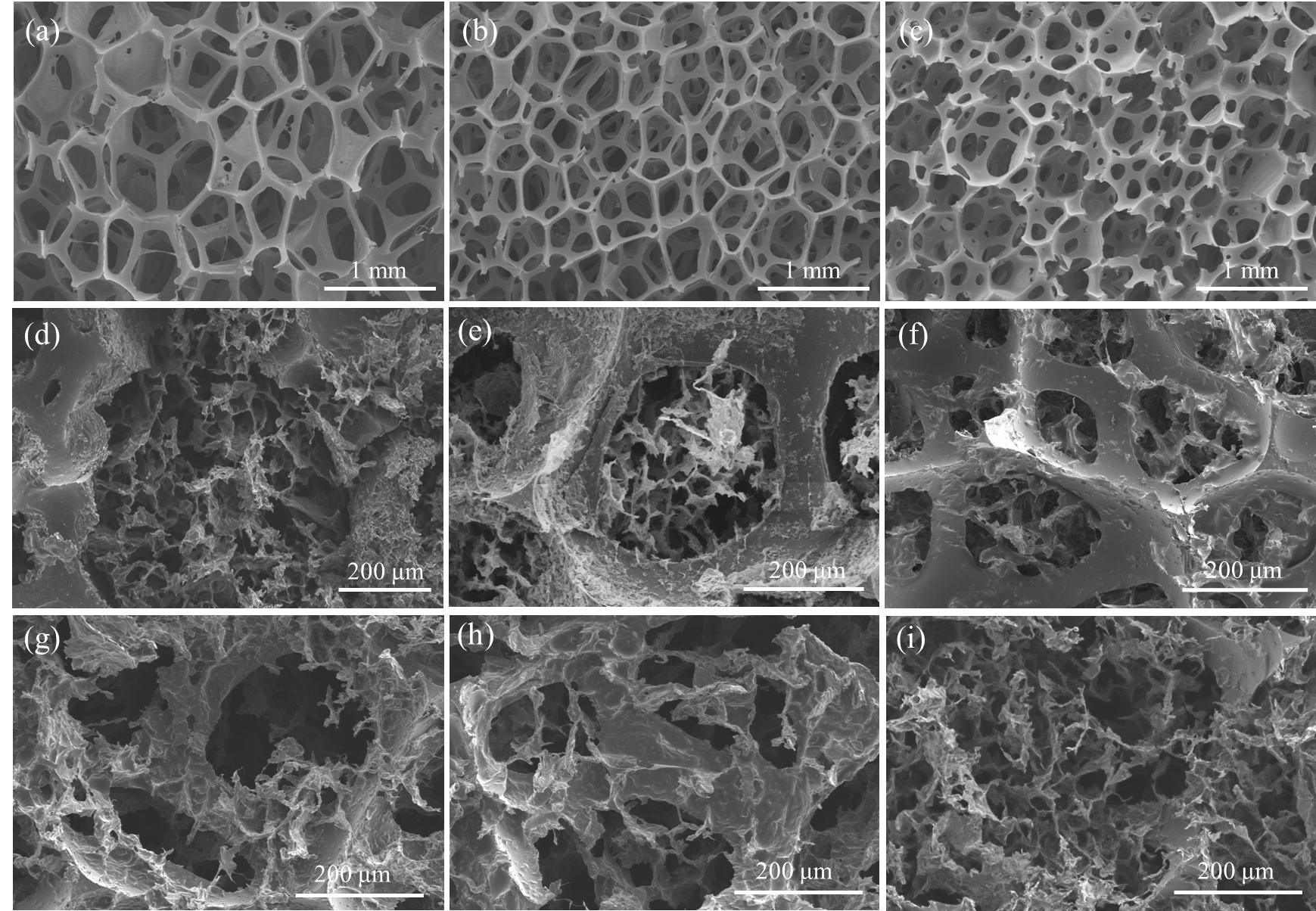


**Figure S21.** PU sponge with different densities: (a) 20kg/m^3^, (b) 30kg/m^3^, (c) 40kg/m^3^; and corresponding MAPUs: (d) MAPU-20, (e) MAPU-30, (f) MAPU-40; MAPU prepared with different aerogel precursor concentrations: (g) MAPU-0, (h) MAPU-1, (i) MAPU-2.


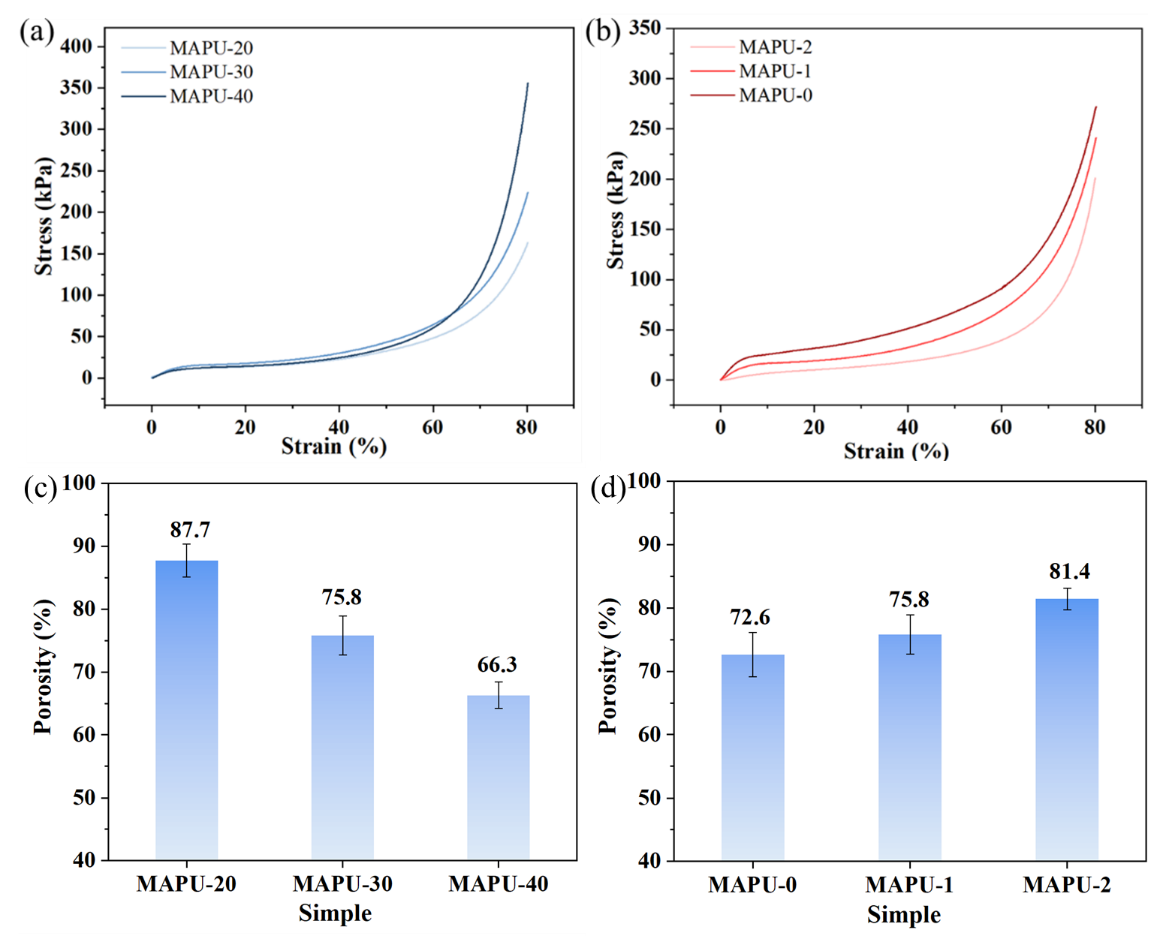


**Figure S22.** (a, b) Compression mechanical curves and (c, d) porosity of different MAPU samples.


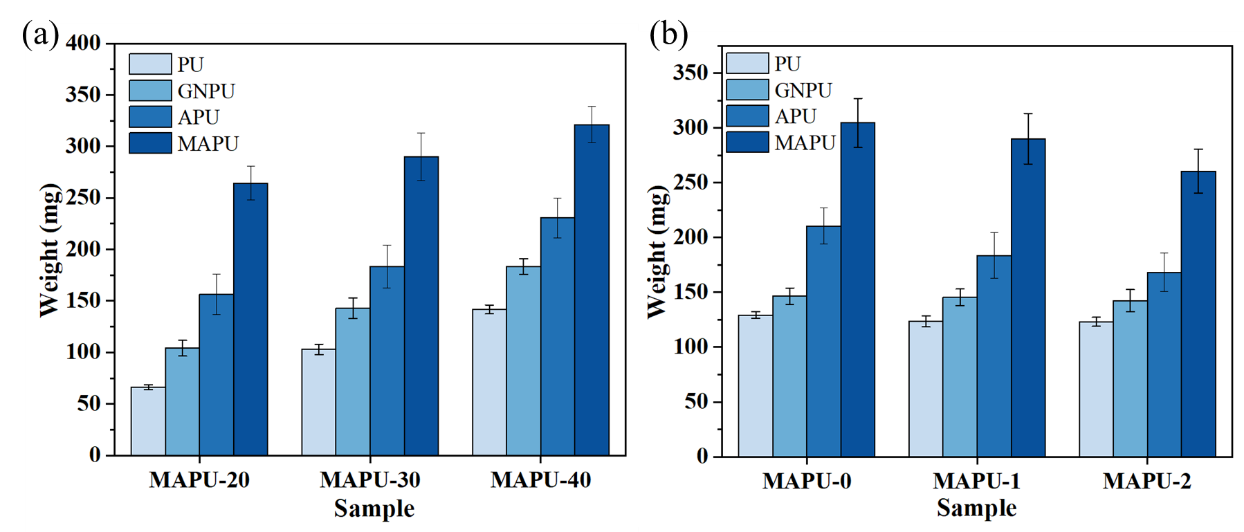


**Figure S23** (a) Weight changes of samples with different density PU sponge substrates during the preparation process, (b) Weight changes of samples with different aerogel loadings during the preparation process


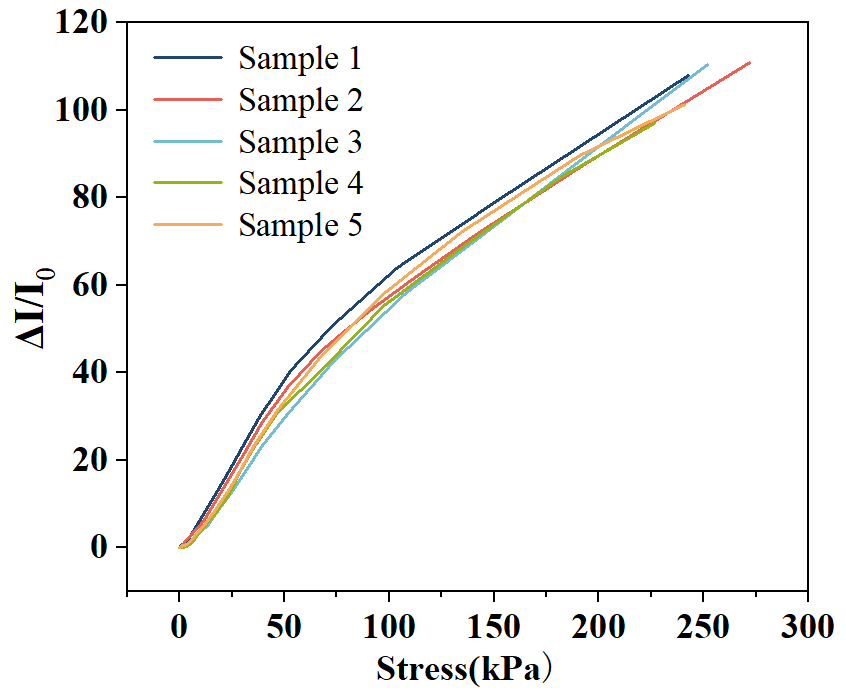


**Figure S24** Piezoresistive curves of MAPU sensors with the same rule, produced in different batches


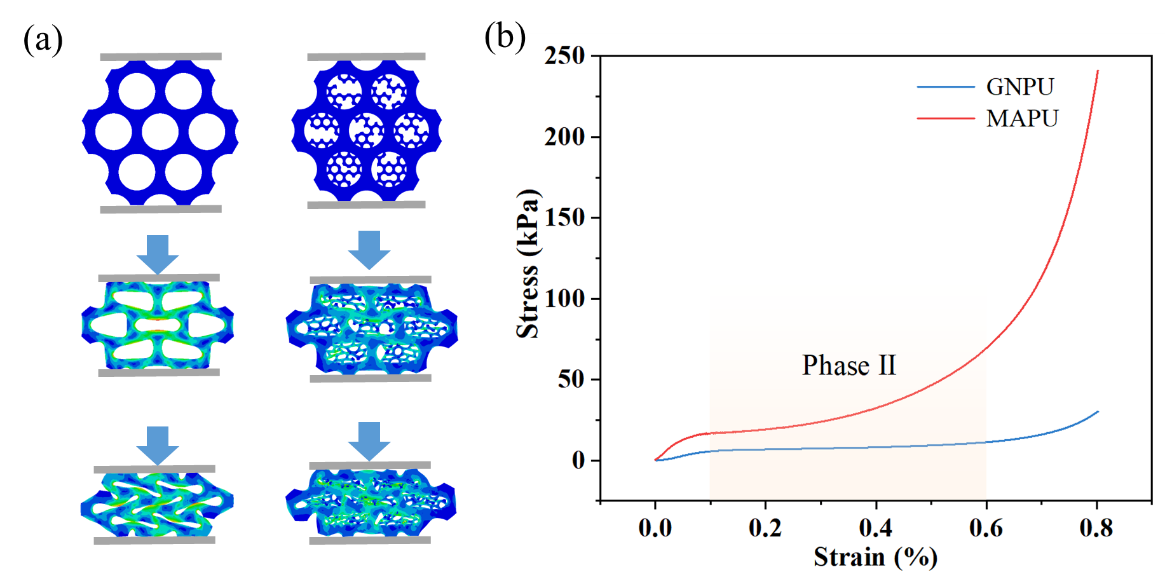


**Figure S25.** (a) Finite element simulations and (b) compressive stress-strain curves of multi- scale network structured MAPU and single-network structured GNPU


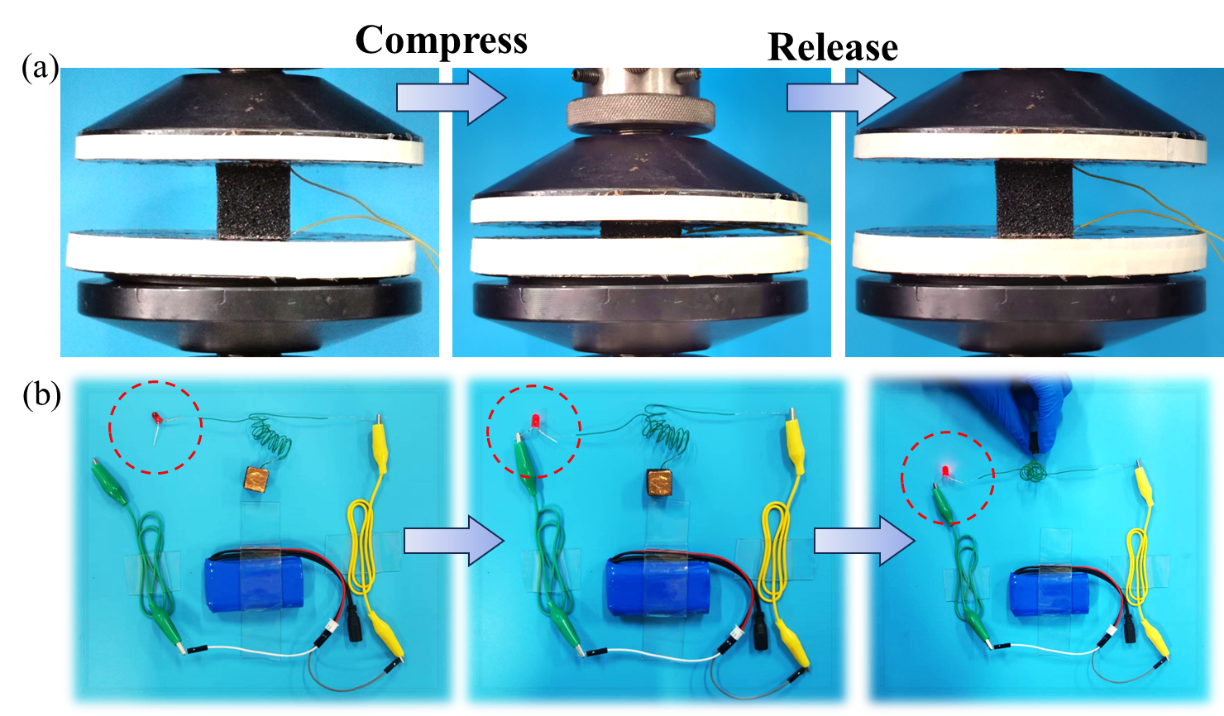


**Figure S26.** (a) MAPU compressed to 80% strain and recovered, (b) comparison of bulb brightness in a circuit connected to MAPU under different conditions: open circuit, closed circuit, and pressing the MAPU.


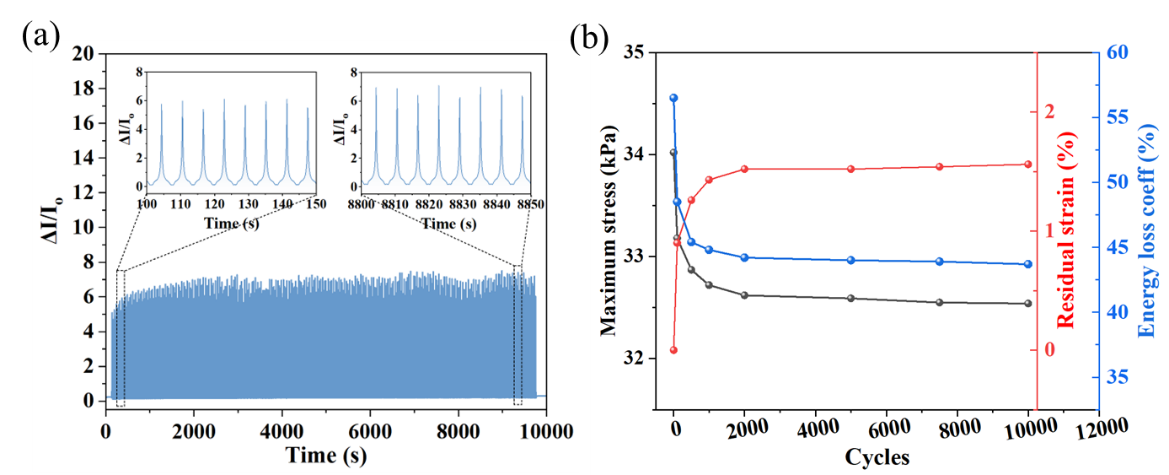


**Figure S27** (a) Electrical signal changes and (b) mechanical property changes during cyclic compression at 50% strain for sponge foam sensors without interpenetrating network structure


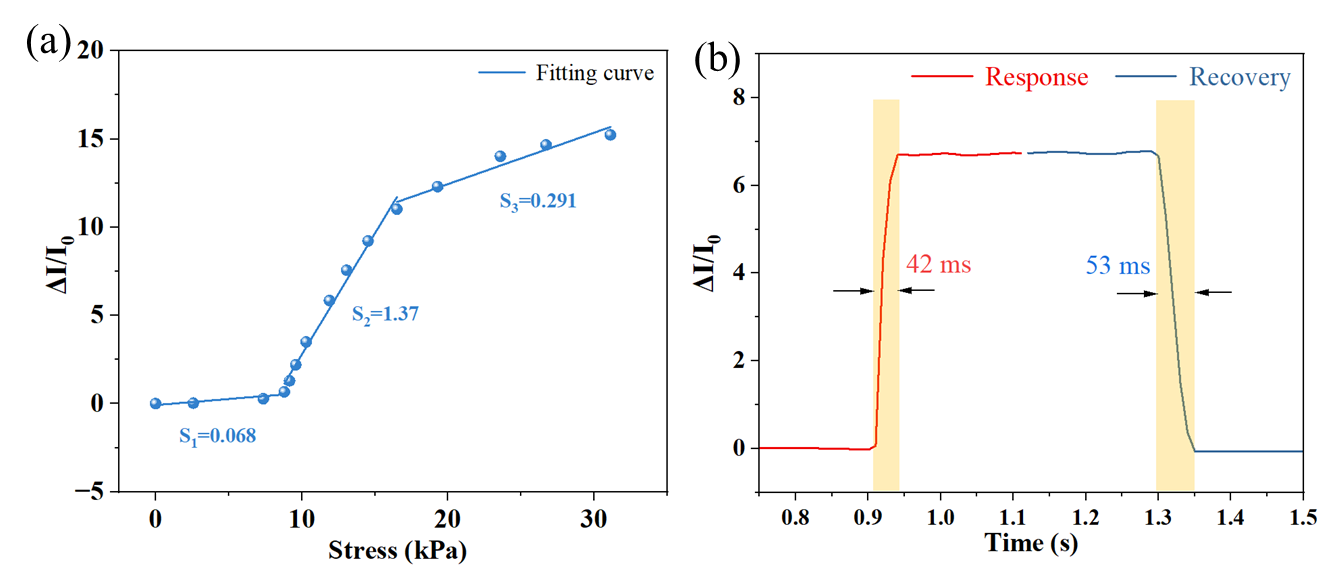


**Figure S28** (a) The sensitivity and working range, and (b) maximum response/recovery time of sponge foam sensors without interpenetrating network structure within the 80% compression strain range


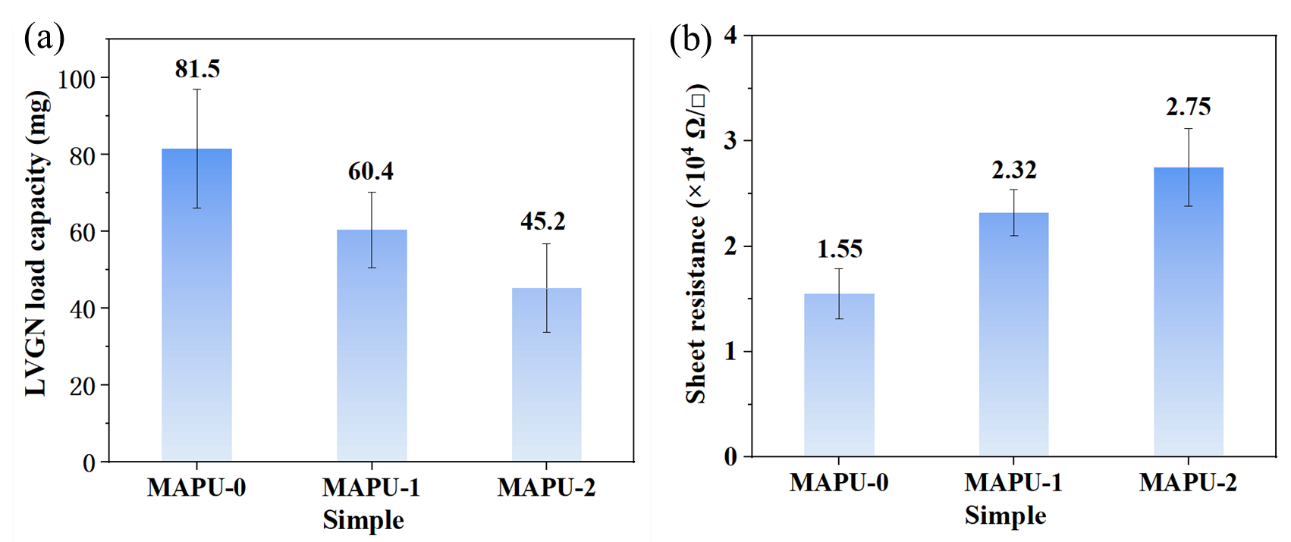


**Figure S29** (a) LVGN loading and (b) sheet resistance of different samples


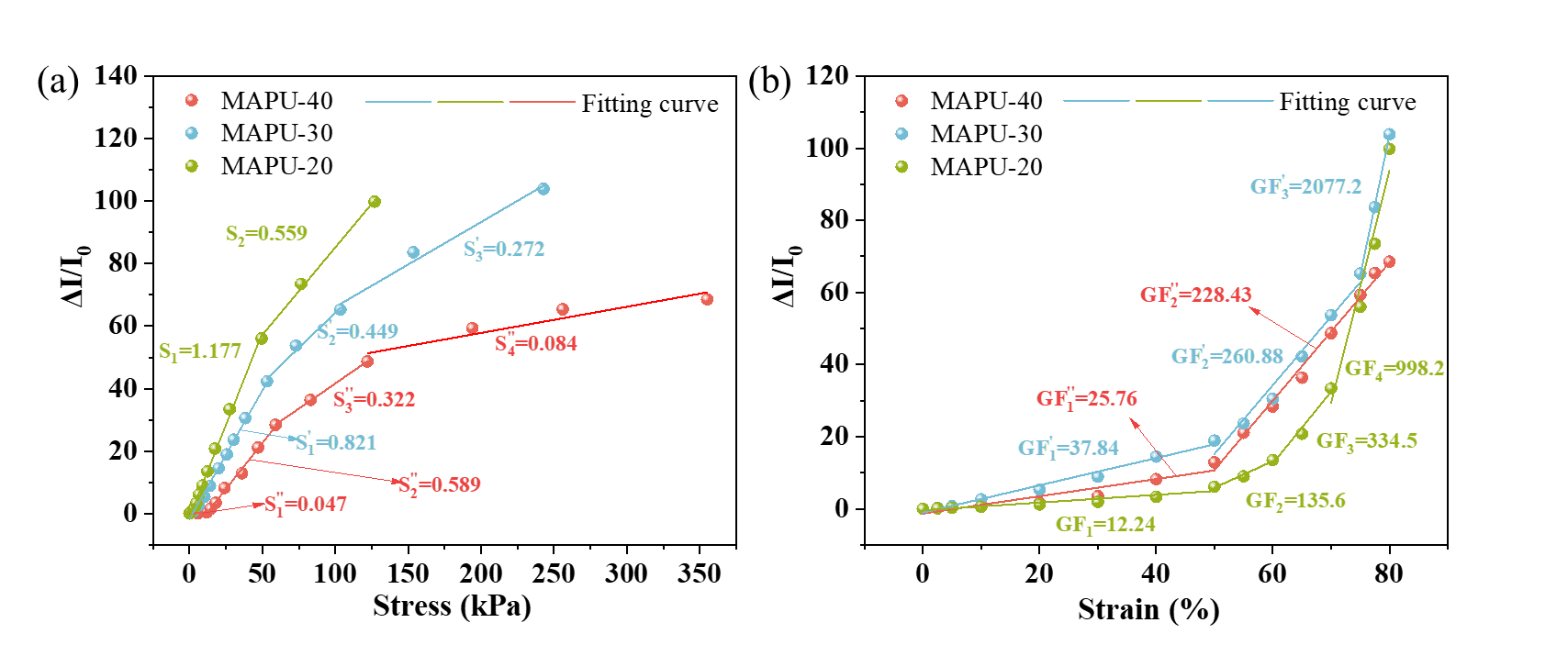


**Figure S30.** (a) Sensitivity and (h) gauge factor of MAPU-20, MAPU-30, and MAPU-40.


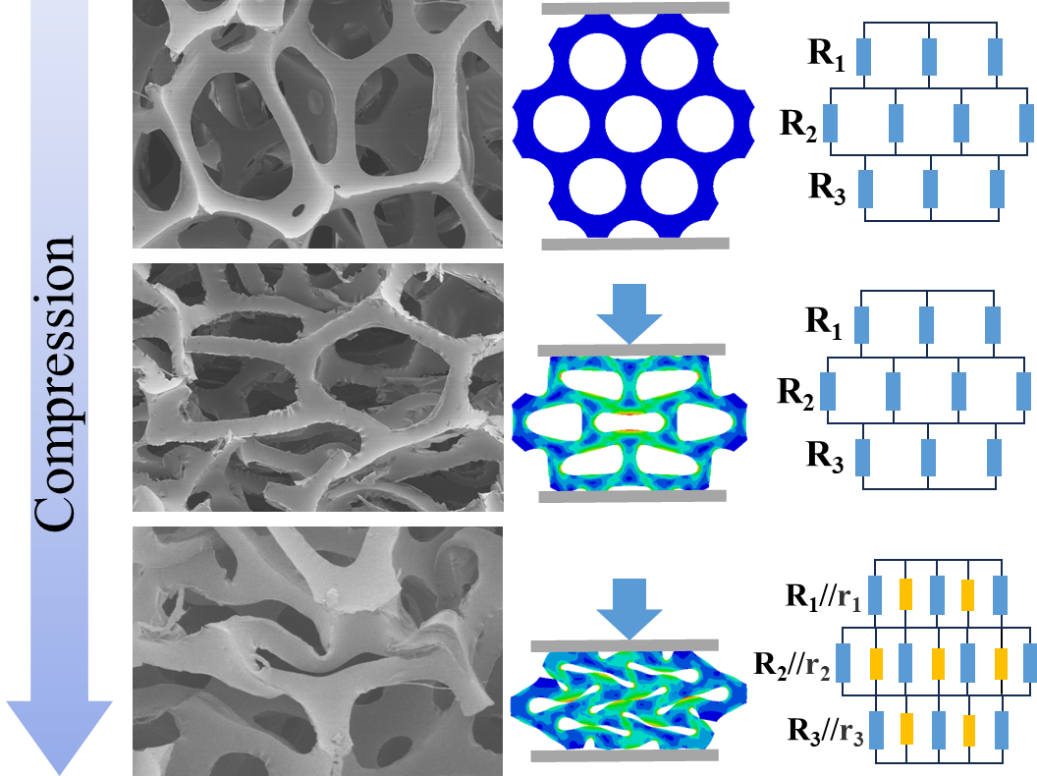


**Figure S31.** Compression process simulation and equivalent circuit of sponge-based sensor without composite aerogel.


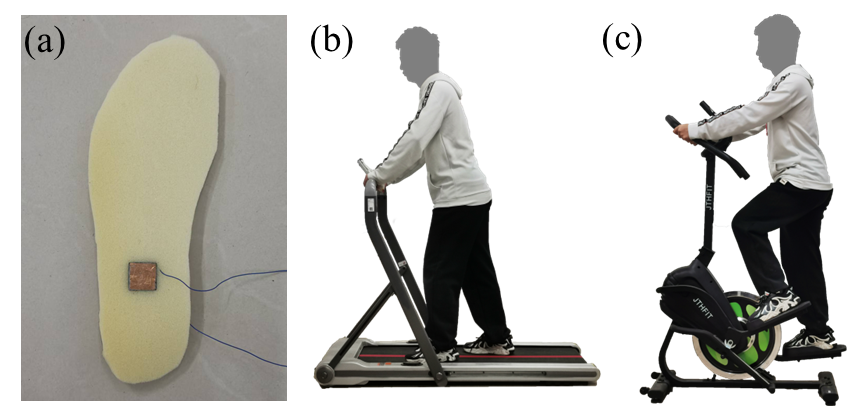


**Figure S32.** (a) Pressure sensing insole embedded with MAPU; plantar pressure test during (b) walking and (c) cycling.


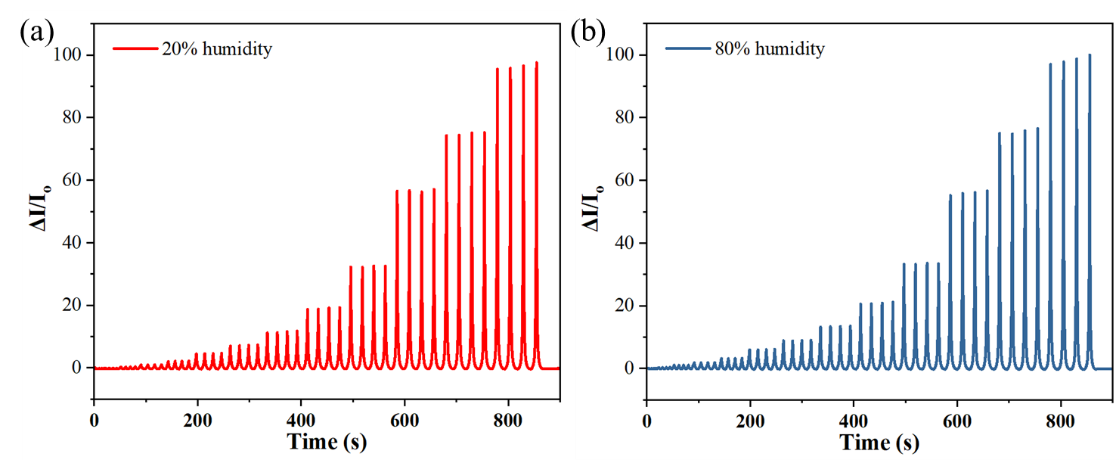


**Figure S33.** Pressure response of MAPU in different relative humidity environments: (a)20%, (b) 80%.


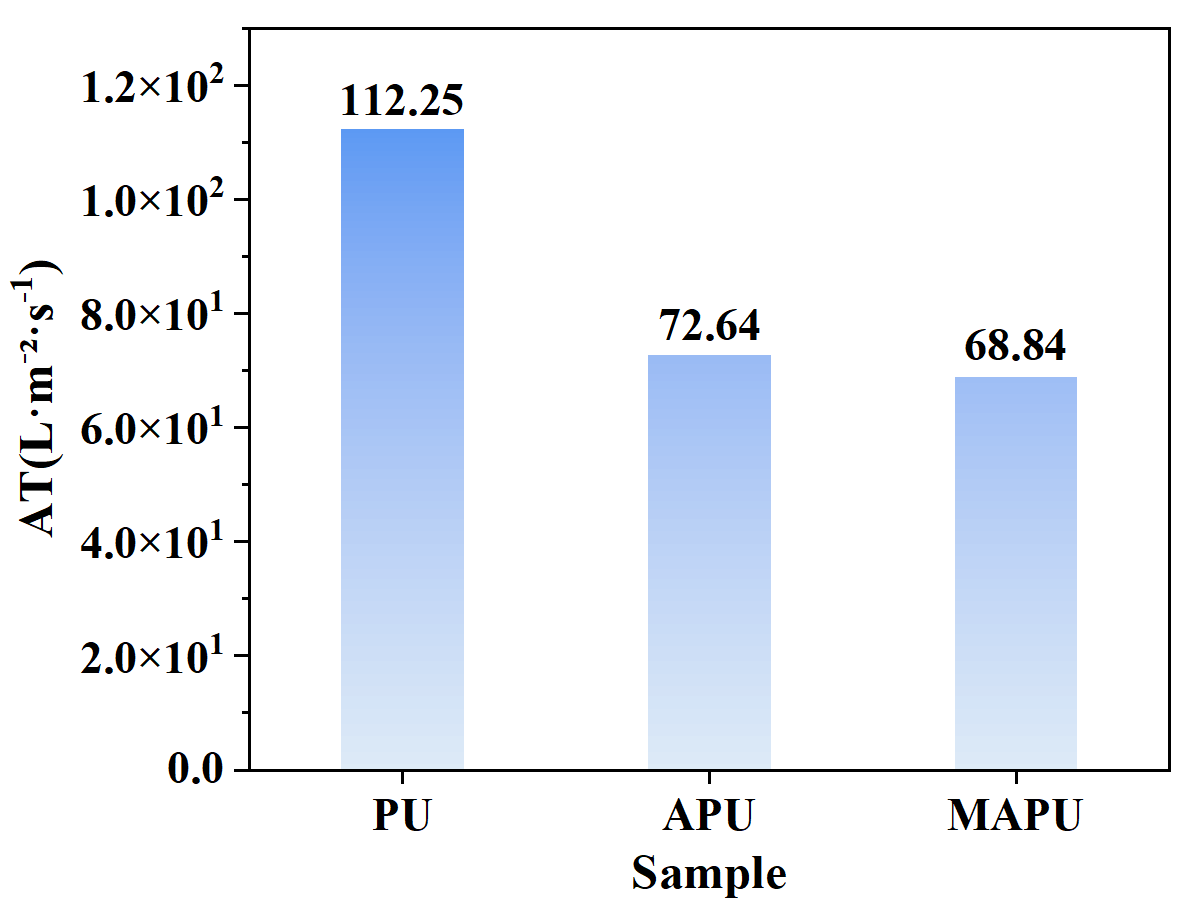


**Figure S34.** Air transmittance of MAPU, APU and PU sponge


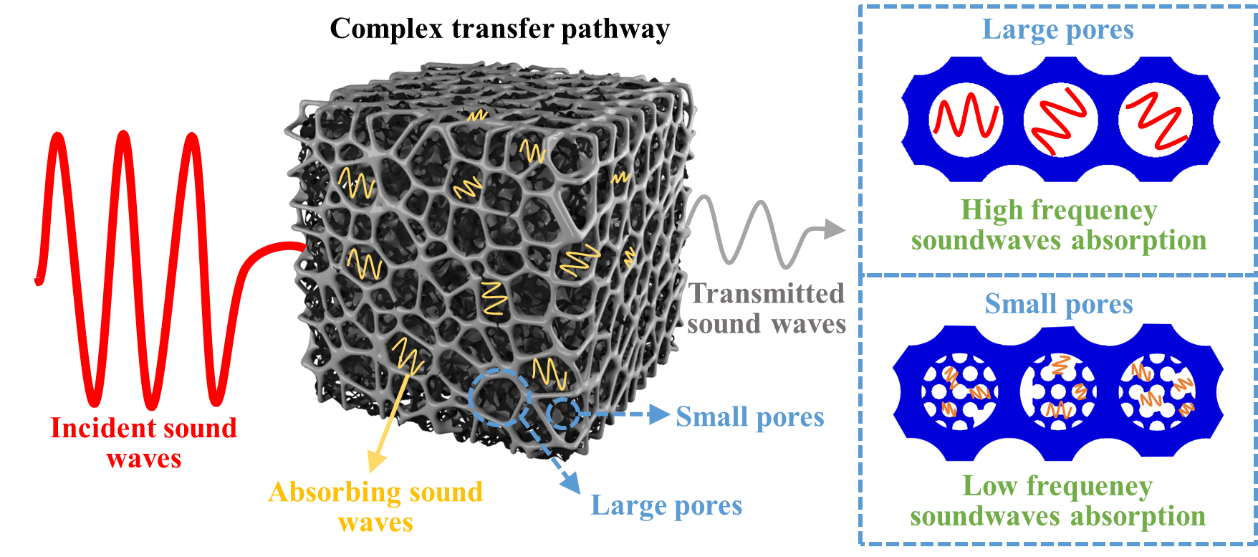


**Figure S35.** The broadband sound absorption mechanism of MAPU.


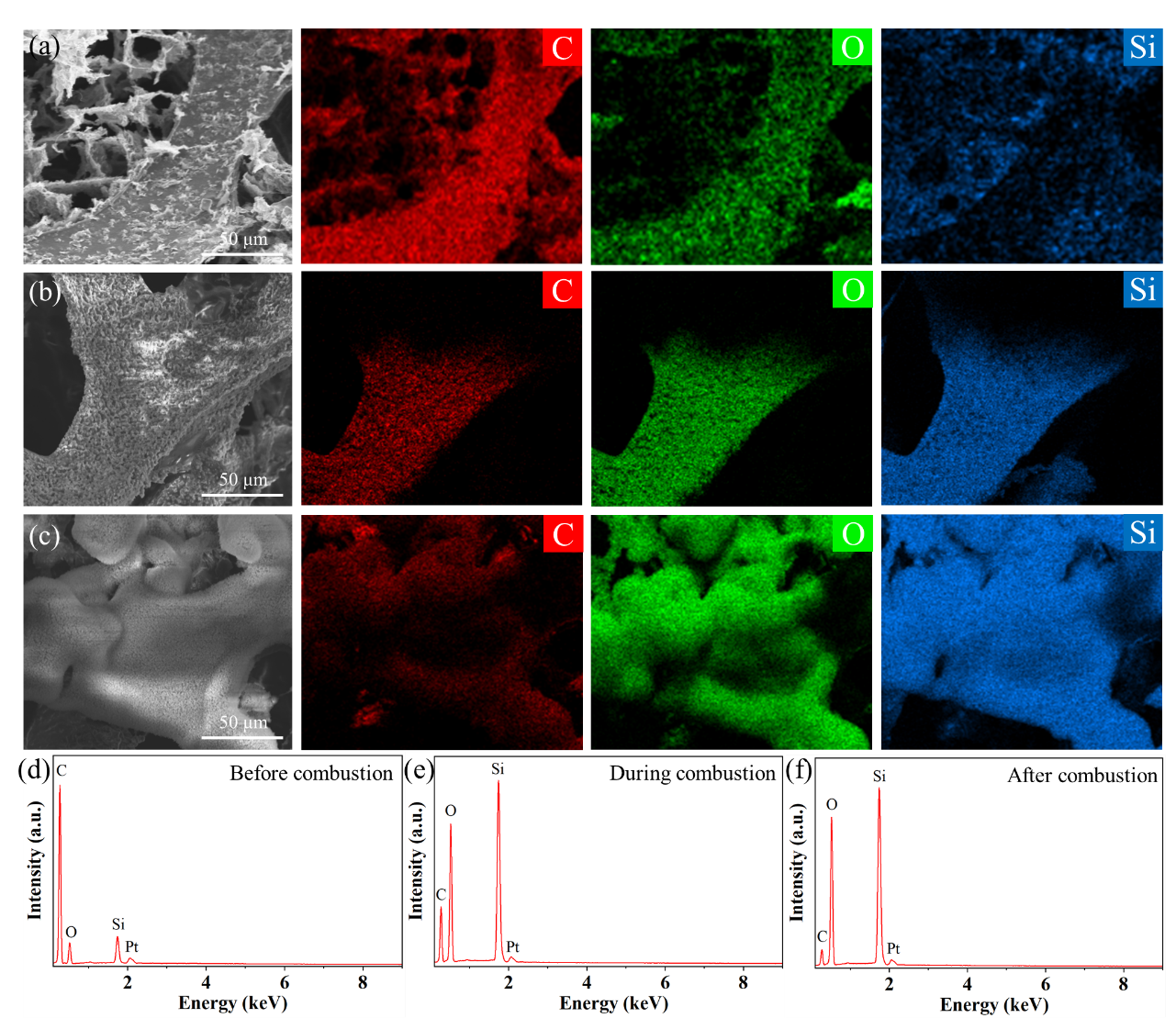


**Figure S36.** EDS images and element energy spectrum of MAPU (a, d) before, (b, e) during and (c, f) after combustion.


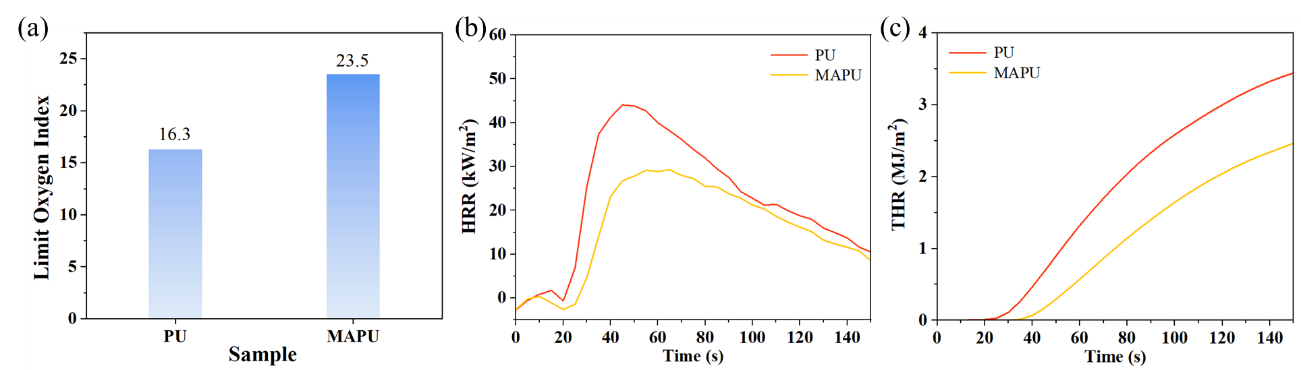


**Figure S37.** Comparison of (a) limit Oxygen Index, (b) combustion heat release rate, and (c) total heat release between MAPU and ordinary PU sponge


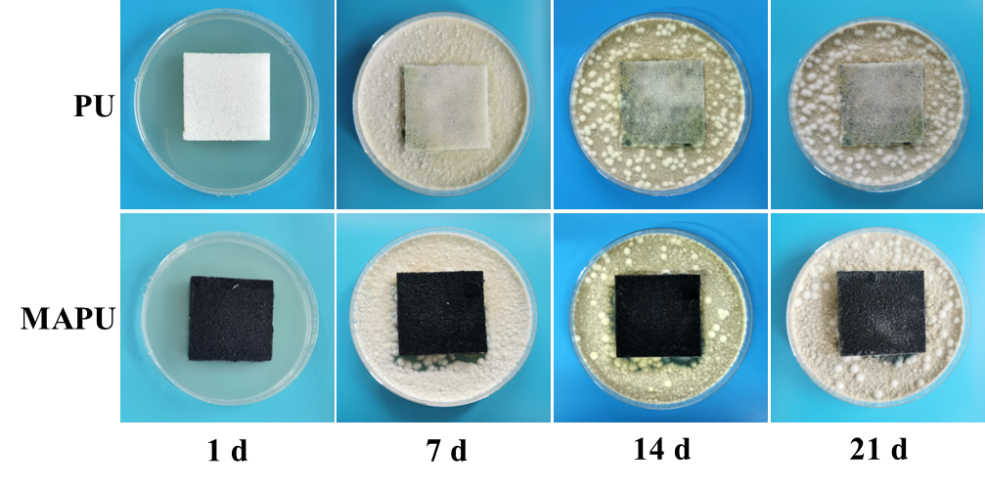


**Figure S38.** Control experiment on mold resistance effect.


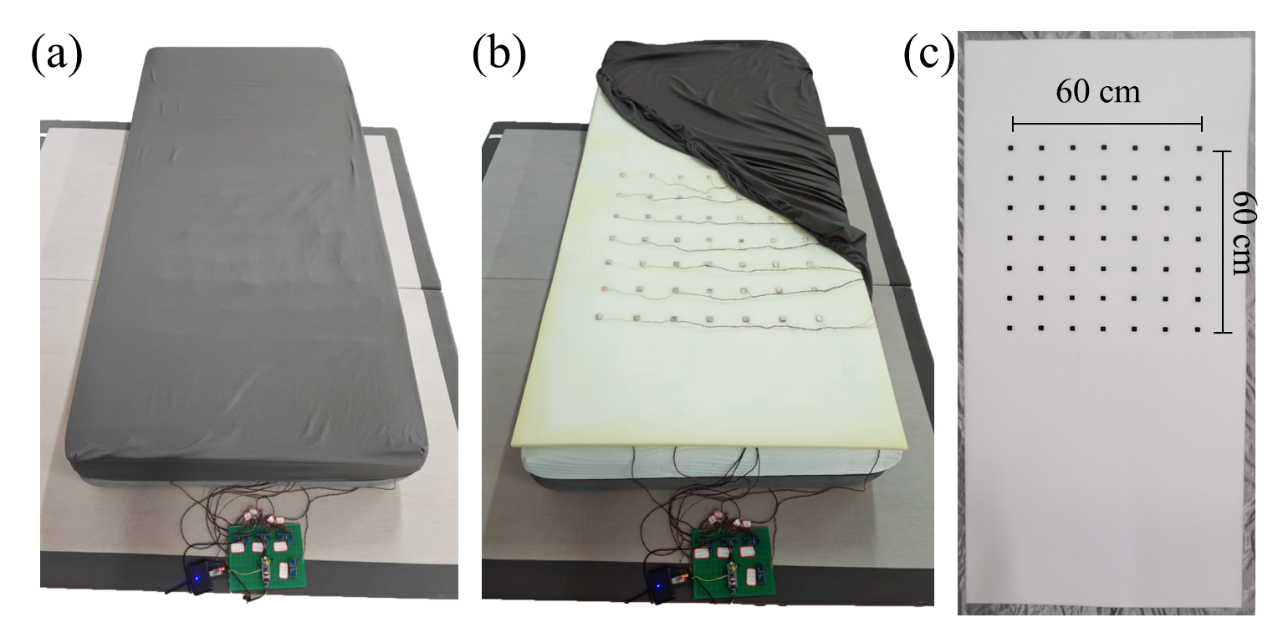


**Figure S39.** (a) Smart mattress; (b) pressure sensing layer; (c) sensor array layout.


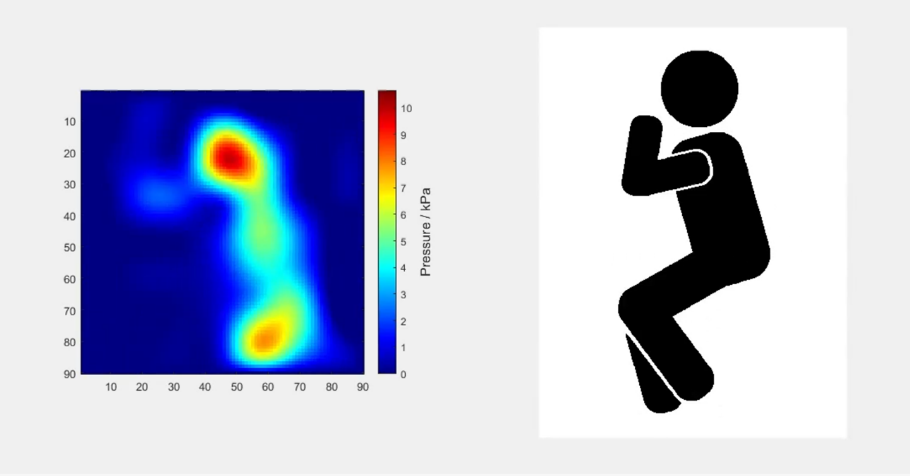


**Figure S40.** GUI including human pressure distribution map and posture recognition.


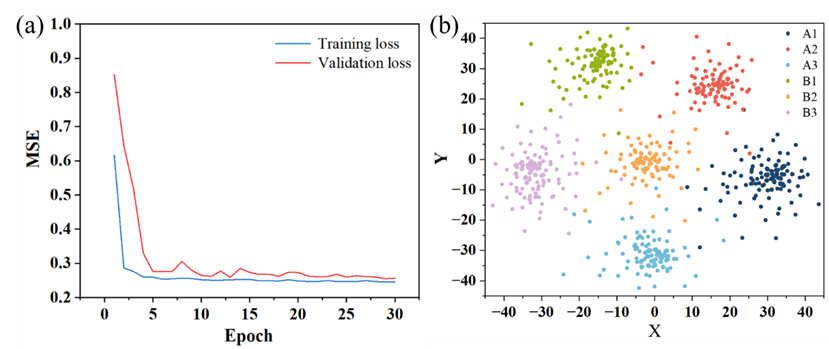


**Figure S41** (a) Training and validation loss curve, (b) clustering diagram of the test set


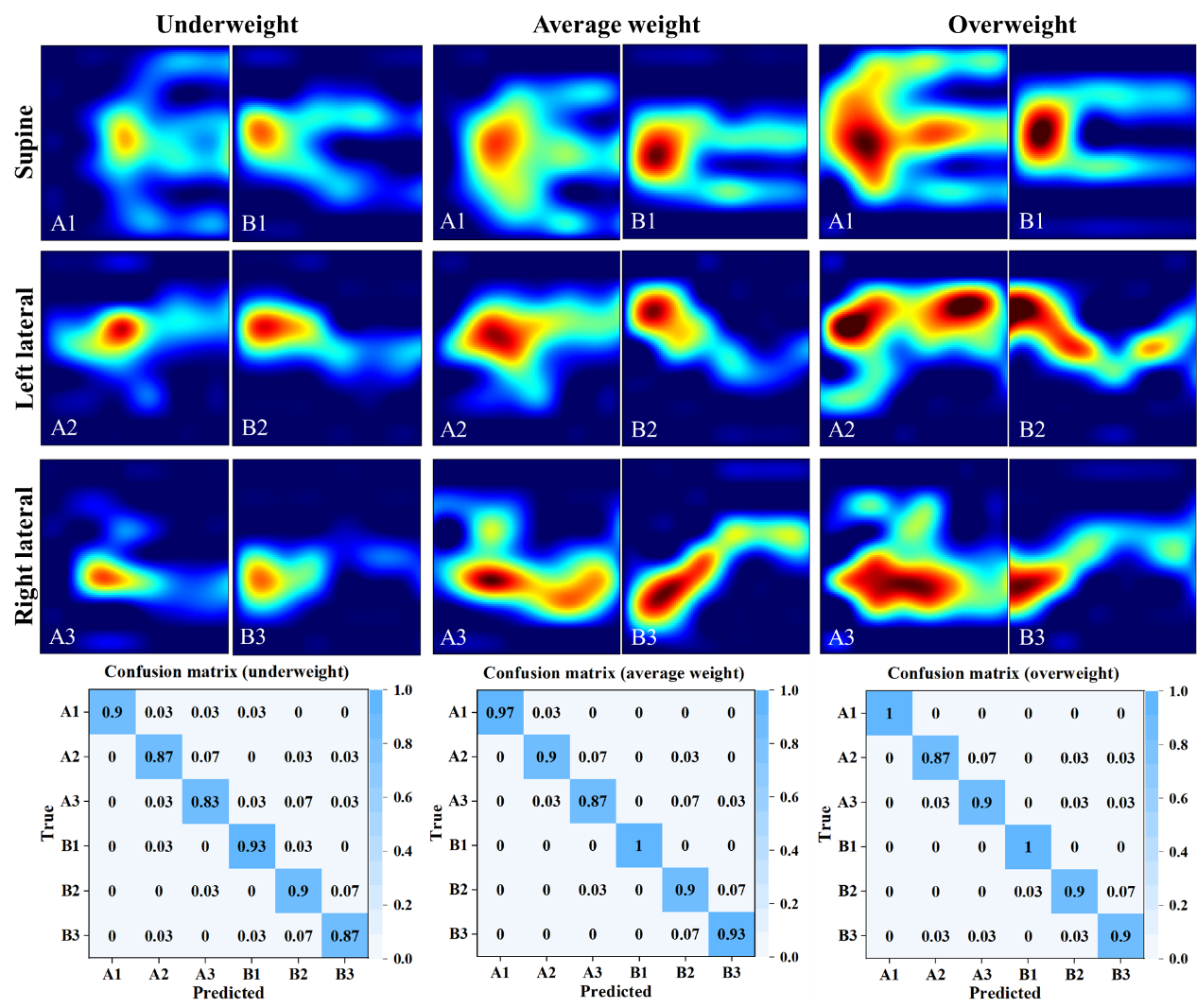


**Figure S42.** Examples of body pressure distribution images and corresponding confusion matrices for subjects with different body types classified according to BMI


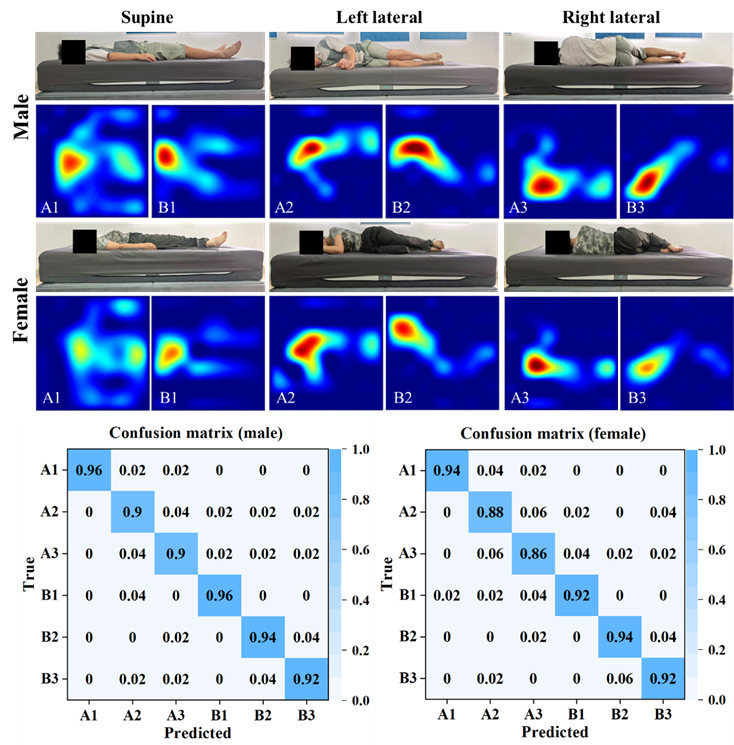


**Figure S43.** Examples of body pressure distribution images and corresponding confusion matrices for male and female subjects


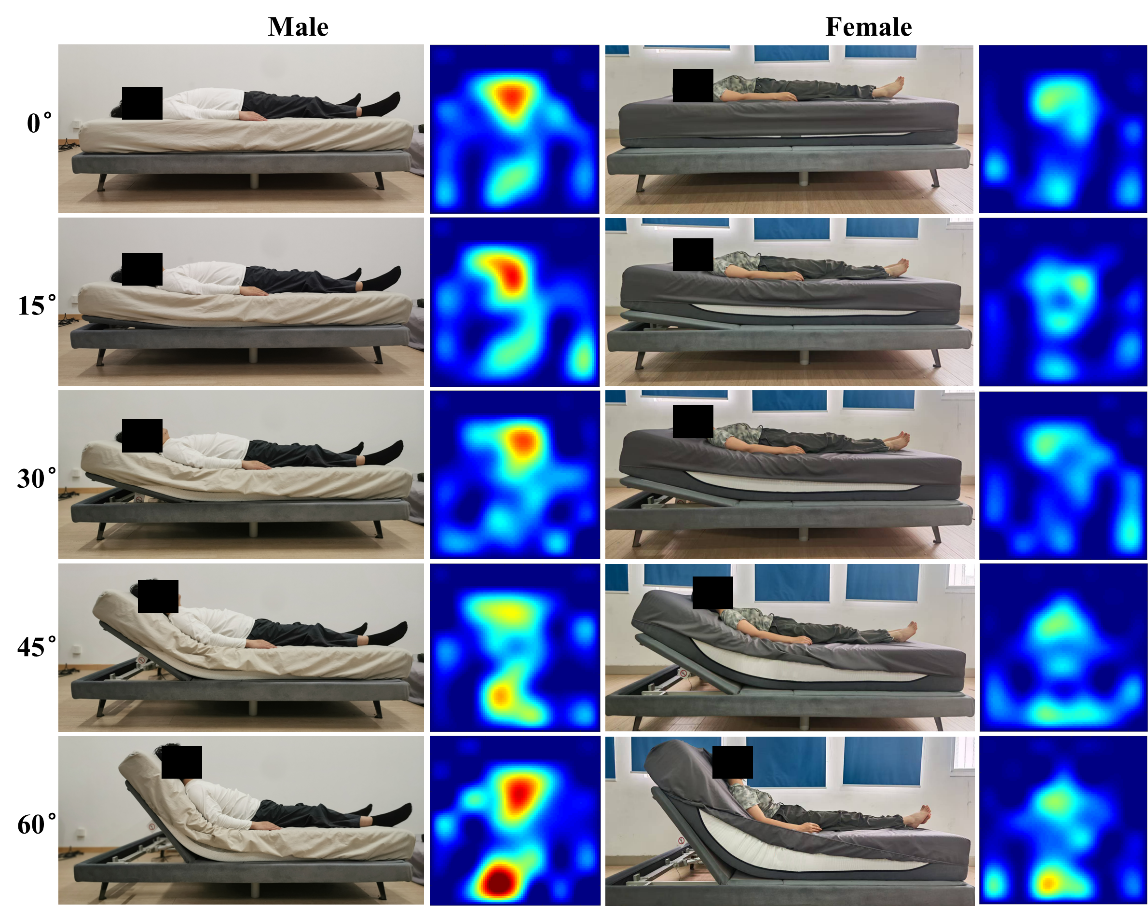


**Figure S44.** Bed angle adjustments and corresponding body pressure distribution maps

**Table S1.** Comparison with data from other similar sensors

| **Type** | **Samples** | **Sensitivity (S)** | **Response time (ms)** | **Maximum strain (%)** | **Operating range (kPa)** | **Stability (Cycles)** | **Ref.** |
| --- | --- | --- | --- | --- | --- | --- | --- |
| **Sponge sensors** | MAPU | 0.821 | 50 | 80 | 0-270 | 30,000 | This study |
|  | MXene@CS@PU | 0.014 | 19 | 85 | 0-245.7 | 5,000 | ^[9]^ |
|  | PDA/PPy/PU | 0.8251 | 120 | 70 | 0-25 | 1,000 | ^[10]^ |
|  | CB@CNF+PU | 0.35 | 100 | 48 | 0-29 | 2,000 | ^[11]^ |
|  | CNT/CB/TPU | 0.1 | 119 | 60 | 0-23.3 | 1,000 | ^[12]^ |
|  | CB@PU | 0.068 | 20 | 60 | 0.091-16.4 | 50,000 | ^[13]^ |
|  | MWNT-rGO@PU | 0.088 | 30 | 50 | 0-2.7 | 5,000 | ^[14]^ |
|  | Graphene/MXene-PDMS @ sponge | 0.053 | 110 | 50 | 0-35 | 2,000 | ^[15]^ |
|  | rGO@PU | 0.0152 | 166 | 80 | 20-1,940 | 10,000 | ^[16]^ |
|  | SWPS | 0.22 | 35 | 60 | 0-156.5 | 1,000 | ^[17]^ |
|  | rGO/ PU | 0.26 | 120 | 95 | 0-10 | 10,000 | ^[18]^ |
|  | PDA/MXene/PU | 0.0389 | 40 | 80 | 0.01-122.5 | 5,000 | ^[19]^ |
|  | GO-AgNF-PI | 0.572 | 100 | 90 | 0-10 | 500 | ^[20]^ |
|  | AgNW/PVDF | 0.014 | 65 | 20 | 0-100 | 10,000 | ^[21]^ |
|  | CNS/TPU | 0.14 | 150 | 60 | 0-18 | 200 | ^[22]^ |
| **Aerogel sensors** | CTA-PoMCF | 0.5 | 32 | 50 | 0-5 | 3,000 | ^[23]^ |
|  | AgNWs/NFC | 3.86 | 180 | 80 | 0.5-1.5 | 10,000 | ^[24]^ |
|  | LCNT/CNF | 2.38 | 100 | 30 | 0.2-9.8 | 1,000 | ^[25]^ |
|  | CNFA | 5.66 | 100 | 90 | 0-28 | 500 | ^[26]^ |
|  | BCSRA | 0.69 | 110 | 70 | 0-9.01 | 1,000 | ^[27]^ |
|  | GO/PVA/MXene | 1.744 | 4.5 | 90 | 0-15 | 5,000 | ^[28]^ |
|  | CNF/PDMS | 0.6 | 30 | 80 | 0-120 | 10,000 | ^[29]^ |
|  | PPy@rGA | 0.9 | 165 | 60 | 0-1 | 10,000 | ^[30]^ |
|  | ZSNFA | 0.262 | 10 | 50 | 0-3 | 1,000 | ^[31]^ |
|  | MXene@ CNTs/CCS | 3.84 | 62 | 50 | 0-80 | 1,000 | ^[32]^ |
|  | PINA@PPy | 0.42 | 300 | 60 | 0-25 | 5,000 | ^[33]^ |
|  | LGA | 3.69 | 50 | 50 | 0-0.6 | 10,000 | ^[34]^ |

**Table S2.** Horizontal combustion test record of MAPU and PU sponge

| Sample | Whether it drips | Burning rate (mm/min) | Damage length (mm) | UL-94 horizontal burning rating |
| --- | --- | --- | --- | --- |
| PU | Yes | 50 | 150 | NR |
| MAPU | No | 13.5 | 65 | HF-2 |

Note: NR indicates that the sample did not receive a rating during testing

**Table S3.** Evaluation criteria for infection values

| Grading value | Description of mildew growth | Mildew growth |
| --- | --- | --- |
| 0 | No growth | No mycelium growth on the surface |
| 1 | Trace growth | A little mycelium on the surface of the specimen, but the infection area is ≤10% |
| 2 | Slight growth | Slight growth of mycelium, the surface of the specimen infected area >10% but ≤30% |
| 3 | Moderate growth | Moderate growth of mycelium, the surface of the specimen infected area >30% but ≤60% |
| 4 | Severe growth | Mycorrhizal serious growth, the surface of the specimen infected area >60% |

**References**

1. Jiao X., Qiu Y., Zhang L., Zhang X., “Comparison of the Characteristic Properties of Reduced Graphene Oxides Synthesized from Natural Graphites with Different Graphitization Degrees.” *RSC Adv.* 2017, *7* (82), 52337–52344. https://doi.org/10.1039/C7RA10809E.

2. Fujimoto H., “A New Estimation Method for the Degree of Graphitization for Random Layer Lattices.” *Carbon* 2010, *48* (12), 3446–3453. https://doi.org/10.1016/j.carbon.2010.05.041.

3. Ungár T., Gubicza J., Ribárik G., Pantea C., Zerda T.W., “Microstructure of Carbon Blacks Determined by X-Ray Diffraction Profile Analysis.” *Carbon* 2002, *40* (6), 929–937. https://doi.org/10.1016/S0008-6223(01)00224-X.

4. Yang J., Illeperuma W., Suo Z., “Inelasticity Increases the Critical Strain for the Onset of Creases on Hydrogels.” *Extreme Mechanics Letters* 2020, *40*, 100966. https://doi.org/10.1016/j.eml.2020.100966.

5. Lu P., Xu J., Wang X., et al., “Gradient Pore Structured Ppy/PDMS Conductive Sponge for Flexible Pressure Sensor.” *Chemical Engineering Journal* 2024, *488*, 151049. https://doi.org/10.1016/j.cej.2024.151049.

6. Bi S., Hou L., Dong W., Lu Y., “Multifunctional and Ultrasensitive-Reduced Graphene Oxide and Pen Ink/Polyvinyl Alcohol-Decorated Modal/Spandex Fabric for High-Performance Wearable Sensors.” *ACS Applied Materials & Interfaces* 2021, *13* (1), 2100–2109. https://doi.org/10.1021/acsami.0c21075.

7. Ma R., Kang B., Cho S., Choi M., Baik S., “Extraordinarily High Conductivity of Stretchable Fibers of Polyurethane and Silver Nanoflowers.” *ACS Nano* 2015, *9* (11), 10876–10886. https://doi.org/10.1021/acsnano.5b03864.

8. Wang X., Tao Y., Pan S., et al., “Biocompatible and Breathable Healthcare Electronics with Sensing Performances and Photothermal Antibacterial Effect for Motion-Detecting.” *npj Flexible Electronics* 2022, *6* (1), 95. https://doi.org/10.1038/s41528-022-00228-x.

9. Li X.-P., Li Y., Li X., et al., “Highly Sensitive, Reliable and Flexible Piezoresistive Pressure Sensors Featuring Polyurethane Sponge Coated with MXene Sheets.” *Journal of Colloid and Interface Science* 2019, *542*, 54–62. https://doi.org/10.1016/j.jcis.2019.01.123.

10. Ren X., Tian Q., Zhu X., et al., “Multi-Scale Closure Piezoresistive Sensor with High Sensitivity Derived from Polyurethane Foam and Polypyrrole Nanofibers.” *Chemical Engineering Journal* 2023, *474*, 145926. https://doi.org/10.1016/j.cej.2023.145926.

11. Xu S., Li X., Sui G., et al., “Plasma Modification of PU Foam for Piezoresistive Sensor with High Sensitivity, Mechanical Properties and Long-Term Stability.” *Chemical Engineering Journal* 2020, *381*, 122666. https://doi.org/10.1016/j.cej.2019.122666.

12. Wang Y., Luo W., Wen Y., et al., “Wearable, Washable Piezoresistive Pressure Sensor Based on Polyurethane Sponge Coated with Composite CNT/CB/TPU.” *Materials Today Physics* 2025, *52*, 101681. https://doi.org/10.1016/j.mtphys.2025.101681.

13. Wu X., Han Y., Zhang X., Zhou Z., Lu C., “Large-Area Compliant, Low-Cost, and Versatile Pressure-Sensing Platform Based on Microcrack-Designed Carbon Black@Polyurethane Sponge for Human–Machine Interfacing.” *Advanced Functional Materials* 2016, *26* (34), 6246–6256. https://doi.org/10.1002/adfm.201601995.

14. Tewari A., Gandla S., Bohm S., McNeill C.R., Gupta D., “Highly Exfoliated MWNT–rGO Ink-Wrapped Polyurethane Foam for Piezoresistive Pressure Sensor Applications.” *ACS Applied Materials & Interfaces* 2018, *10* (6), 5185–5195. https://doi.org/10.1021/acsami.7b15252.

15. Sang S., Jing Z., Cheng Y., et al., “Graphene and MXene-Based Sponge Pressure Sensor Array for Rectal Model Pressure Detection.” *Macromolecular Materials and Engineering* 2021, *306* (10), 2100251. https://doi.org/10.1002/mame.202100251.

16. Lü X., Yu T., Meng F., Bao W., “Wide-Range and High-Stability Flexible Conductive Graphene/Thermoplastic Polyurethane Foam for Piezoresistive Sensor Applications.” *Advanced Materials Technologies* 2021, *6* (10), 2100248. https://doi.org/10.1002/admt.202100248.

17. Song H., Ma J., Li C., et al., “Design of a Stable Wearable Piezoresistive Sensor with a Laminated Pattern for Simultaneous Anti-Wetting and Self-Power.” *Chemical Engineering Journal* 2024, *481*, 148346. https://doi.org/10.1016/j.cej.2023.148346.

18. Yao H.-B., Ge J., Wang C.-F., et al., “A Flexible and Highly Pressure-Sensitive Graphene–Polyurethane Sponge Based on Fractured Microstructure Design.” *Advanced Materials* 2013, *25* (46), 6692–6698. https://doi.org/10.1002/adma.201303041.

19. Chen Q., Gao Q., Wang X., Schubert D.W., Liu X., “Flexible, Conductive, and Anisotropic Thermoplastic Polyurethane/Polydopamine /MXene Foam for Piezoresistive Sensors and Motion Monitoring.” *Composites Part A: Applied Science and Manufacturing* 2022, *155*, 106838. https://doi.org/10.1016/j.compositesa.2022.106838.

20. Khan F.A., Ajmal C.M., Bae S., et al., “Silver Nanoflower Decorated Graphene Oxide Sponges for Highly Sensitive Variable Stiffness Stress Sensors.” *Small* 2018, *14* (24), 1800549. https://doi.org/10.1002/smll.201800549.

21. Jing M., Zhou J., Zhang P., et al., “Porous AgNWs/Poly(Vinylidene Fluoride) Composite-Based Flexible Piezoresistive Sensor with High Sensitivity and Wide Pressure Ranges.” *ACS Applied Materials & Interfaces* 2022, *14* (49), 55119–55129. https://doi.org/10.1021/acsami.2c17879.

22. Huang W., Dai K., Zhai Y., et al., “Flexible and Lightweight Pressure Sensor Based on Carbon Nanotube/Thermoplastic Polyurethane-Aligned Conductive Foam with Superior Compressibility and Stability.” *ACS Applied Materials & Interfaces* 2017, *9* (48), 42266–42277. https://doi.org/10.1021/acsami.7b16975.

23. Wang G., Liu X., Song Z., et al., “Multifunctional Flexible Pressure Sensor Based on a Cellulose Fiber-Derived Hierarchical Carbon Aerogel.” *ACS Applied Electronic Materials* 2023, *5* (3), 1581–1591. https://doi.org/10.1021/acsaelm.2c01628.

24. Cheng R., Zeng J., Wang B., et al., “Ultralight, Flexible and Conductive Silver Nanowire/Nanofibrillated Cellulose Aerogel for Multifunctional Strain Sensor.” *Chemical Engineering Journal* 2021, *424*, 130565. https://doi.org/10.1016/j.cej.2021.130565.

25. Du X., Chen Q., Zhou Q., et al., “3D Printing Lignin Carbonized Nanotube and Cellulose Nano Fiber Aerogel for Wearable Pressure Sensors.” *Composites Science and Technology* 2025, *260*, 110976. https://doi.org/10.1016/j.compscitech.2024.110976.

26. Wang B., Yin X., Cheng R., et al., “Compressible, Superelastic and Fatigue Resistant Carbon Nanofiber Aerogels Derived from Bacterial Cellulose for Multifunctional Piezoresistive Sensors.” *Carbon* 2022, *199*, 318–328. https://doi.org/10.1016/j.carbon.2022.08.006.

27. Wang J., Dong Z., Li J., et al., “Highly Compressible Lamellar Graphene/Cellulose/Sodium Alginate Aerogel via Bidirectional Freeze-Drying for Flexible Pressure Sensor.” *International Journal of Biological Macromolecules* 2025, *297*, 139867. https://doi.org/10.1016/j.ijbiomac.2025.139867.

28. Chen T., Yang G., Li Y., et al., “Temperature-Adaptable Pressure Sensors Based on MXene-Coated GO Hierarchical Aerogels with Superb Detection Capability.” *Carbon* 2022, *200*, 47–55. https://doi.org/10.1016/j.carbon.2022.08.002.

29. Dai S.-W., Gu Y.-L., Zhao L., et al., “Bamboo-Inspired Mechanically Flexible and Electrically Conductive Polydimethylsiloxane Foam Materials with Designed Hierarchical Pore Structures for Ultra-Sensitive and Reliable Piezoresistive Pressure Sensor.” *Composites Part B: Engineering* 2021, *225*, 109243. https://doi.org/10.1016/j.compositesb.2021.109243.

30. Wei H., Li A., Kong D., et al., “Polypyrrole/Reduced Graphene Aerogel Film for Wearable Piezoresisitic Sensors with High Sensing Performances.” *Advanced Composites and Hybrid Materials* 2021, *4* (1), 86–95. https://doi.org/10.1007/s42114-020-00201-0.

31. Xiao W., Lu L., Xu Z., et al., “A Superelastic Ceramic Aerogel for Flexible Pressure Sensor in Harsh Environment.” *Composites Part B: Engineering* 2025, *292*, 112110. https://doi.org/10.1016/j.compositesb.2024.112110.

32. Yang Z., Li H., Zhang S., Lai X., Zeng X., “Superhydrophobic MXene@carboxylated Carbon Nanotubes/Carboxymethyl Chitosan Aerogel for Piezoresistive Pressure Sensor.” *Chemical Engineering Journal* 2021, *425*, 130462. https://doi.org/10.1016/j.cej.2021.130462.

33. Lin J., Li J., Li W., et al., “Multifunctional Polyimide Nanofibrous Aerogel Sensor for Motion Monitoring and Airflow Perception.” *Composites Part A: Applied Science and Manufacturing* 2024, *178*, 108003. https://doi.org/10.1016/j.compositesa.2023.108003.

34. Min P., Li X., Liu P., et al., “Rational Design of Soft Yet Elastic Lamellar Graphene Aerogels via Bidirectional Freezing for Ultrasensitive Pressure and Bending Sensors.” *Advanced Functional Materials* 2021, *31* (34), 2103703. https://doi.org/10.1002/adfm.202103703.
